# MicroRNAs emerging coordinate with placental mammals alter pathways in endometrial epithelia important for endometrial function

**DOI:** 10.1016/j.isci.2023.106339

**Published:** 2023-03-04

**Authors:** Laura Hume, Jessica C. Edge, Haidee Tinning, Dapeng Wang, Alysha S. Taylor, Vladimir Ovchinnikov, Annika V. Geijer-Simpson, Pavle Vrljicak, Jan J. Brosens, Emma S. Lucas, Nigel A.B. Simpson, Jayne Shillito, Karen Forbes, Mary J. O’Connell, Niamh Forde

**Affiliations:** 1 Discovery and Translational Sciences Department, Leeds Institute of Cardiovascular and Metabolic Medicine, Faculty of Medicine and Health, University of Leeds, Leeds, LS2 9JT, UK; 2 LeedsOmics, University of Leeds, Leeds, UK; 3 School of Biology, Faculty of Biological Sciences, University of Leeds, Leeds, LS2 9JT, UK; 4 Division of Biomedical Sciences, Clinical Sciences Research Laboratories, Warwick Medical School, University of Warwick, Coventry CV2 2DX, UK; 5 Tommy’s National Centre for Miscarriage Research, University Hospital Coventry and Warwickshire, Coventry CV2 2DX, UK; 6 Department of Women’s and Children’s Health, School of Medicine, University of Leeds, Leeds LS2 9JT, UK; 7 Leeds Teaching Hospitals Trust, Beckett St, Leeds LS9 7TF, UK; 8 School of Life Sciences, Faculty of Medicine and Health Sciences, University of Nottingham, Nottingham, NG7 2RD, UK

**Keywords:** Biological sciences, Molecular biology, Developmental biology, Embryology

## Abstract

We tested the hypothesis that conserved placental mammal-specific microRNAs and their targets facilitate endometrial receptivity to implantation. Expression of miR-340-5p, -542-3p, and -671-5p was regulated by exposure of endometrial epithelial cells to progesterone (10 μg/ml) for 24 h coordinate with 1,713 of their predicted targets. Proteomic analysis of cells transfected with miRNA mimic/inhibitor (48 h: n = 3) revealed 1,745 proteins altered by miR-340-5p (mimic; 1,369, inhibitor; 376) of which 171 were predicted targets and P4-regulated. MiR-542-3p altered 2,353 (mimic; 1,378, inhibitor; 975) 100 which were mirDB predicted, including 46 P4-regulated. MiR-671-5p altered 1,744 proteins (mimic; 1,252, inhibitor; 492) 95 of which were predicted targets and 46 P4-regulated. All miRNAs were detected in luteal phase endometrial biopsies, irrespective of pregnancy outcomes. miR-340-5p expression increased in biopsies from individuals suffering previous and subsequent miscarriage compared to those with subsequent live birth. Dysfunction of these miRNAs and their targets contribute to endometrial-derived recurrent pregnancy loss.

## Highlights

• Progesterone upregulates miR-340-5p, -542-3p and -671-5p in endometrial epithelium
• Progesterone alters transcripts involved in proliferation and epithelial development
• Over- or under-expressing these microRNAs results in altered abundance of proteins
• Functions of altered proteins are involved in implantation and uterine receptivity

## Graphical abstract

**Figure:**
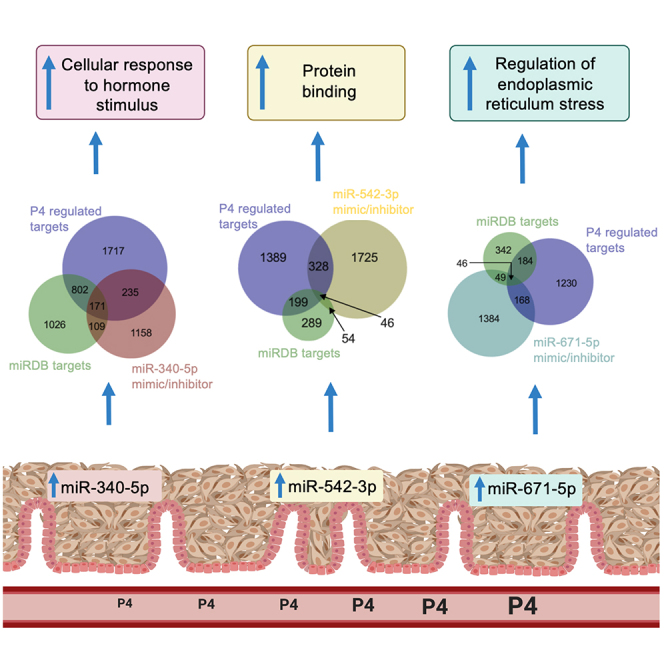

## Introduction

Successful pregnancy in all placental mammals, including humans, is contingent on a carefully orchestrated series of events, including fertilisation between competent gametes, appropriate embryo development, successful implantation, and the establishment of a functional placenta. Perturbations at any of these key developmental points, such as chromosomal abnormalities in the embryo, are known to result in pregnancy loss. However, the contribution of an inadequately regulated endometrium is considerably underexplored. In humans, the uterine endometrium is a heterogeneous tissue composed of luminal and glandular epithelial cells, underlying fibroblast-like stromal cells, and populations of immune cells, vasculature and stem cells.^1^ Spatial and temporal responsiveness of these cells to hormonal cues and other signaling molecules throughout the menstrual cycle facilitates rapid proliferation and differentiation of the endometrial lining to prepare for successful implantation. The appropriate physiological response of the endometrium depends not only on the cellular response to systemic cues, such as circulating estradiol and progesterone (P4) levels, but also on the inter- and intra-cellular communication between different cell types.

Inappropriate remodeling of the endometrium, both before and at the time of implantation, can lead to pregnancy failure such as miscarriage even when a developmentally competent embryo implants.^2^^,^^3^^,^^4^ Indeed, miscarriage can affect up to 1 in 6 pregnancies.^5^ The dynamic transcriptional responses of the endometrium during the menstrual cycle and implantation (reviewed by^6^) have been investigated in detail in relation to how they contribute to miscarriage.^6^^,^^7^^,^^8^ However, the regulatory signaling networks responsible for establishing a receptive endometrium to facilitate implantation, and their dysregulation in recurrent pregnancy loss (RPL), have yet to be determined.

Given their flexible and dynamic regulatory affects, miRNAs may be key in these processes. Once processed from pri- and pre-miRNA forms, these small RNA molecules are usually 22 nucleotides long and bind primarily to the 3′UTR of mRNAs and either suppress the initiation of translation or promote the degradation of the mRNA itself.^9^ They play key roles in inter-cellular communication as components of extracellular vesicles (EVs) or modify direct cargo loading to EVs.^9^ Some candidate microRNAs (miRNAs) that are altered in the endometrium have been reported to relate to early miscarriage.^6^^,^^7^^,^^8^ The expression of miRNAs varies dramatically and dynamically during pregnancy,^10^ with dysregulation of specific miRNAs also having been associated with other pregnancy disorders including preeclampsia and intrauterine growth restriction.^11^^,^^12^ However, conflicting data between studies as well as cell-specific patterns of expression and regulation of miRNA mean we do not fully understand their role in normal and dysfunctional endometria.

The origin of placental mammals is synonymous with the emergence of placentation, lactation and also of hair. This burst of morphological innovation was mediated by a combination of novel and repurposed genomic elements (both protein coding and non-protein coding), regulatory mechanisms, signaling pathways, cells and tissues.^13^^,^^14^^,^^15^ Indeed, bilateral signaling between fetal and maternal tissues is key to establishment of the mammalian placenta. In humans in particular, invasion of the maternal endometrium by the fetal trophoblast required coordinate specialization of these distinct tissues with the co-evolution of the underlying molecular signaling networks facilitating these interactions and communications.^13^ Recent data using an evolutionary approach has identified a key role for the transcription factor, HAND2, and its target genes as important in the pathogenesis of pre-term birth and gestation length.^16^^,^^17^ We have adopted a similar strategy, leading to the identification of 13 miRNA families (17 microRNA genes) that arose coordinate with placental mammals and have been retained in all modern placental mammals.^18^ These miRNAs are detectable in the endometrial epithelium and their expression is altered in a species-specific manner by proteins known to facilitate signaling between the embryo and endometrium.^19^^,^^20^

Given that (1) the endometrium and the trophoblast had to co-evolve regulatory networks to support implantation and early pregnancy success, (2) miRNAs can be important regulators in establishing these types of networks and (3) miRNA (and their targets) are regulated by molecules important to establish receptivity to implantation, we tested the hypothesis that these mammal-specific miRNA genes and their targets are regulated in the endometrium to facilitate receptivity to implantation. We specifically focus on the endometrial epithelium as this is where uterine receptivity is established and also the site of the first biophysical interaction between the embryo and endometrium. Furthermore, we propose that dysregulated expression of these miRNAs in the endometrium may be a feature in women with early pregnancy loss.

## Results

### Progesterone alters selected miRNAs and predicted targets *in vitro*

**Figure 1:**
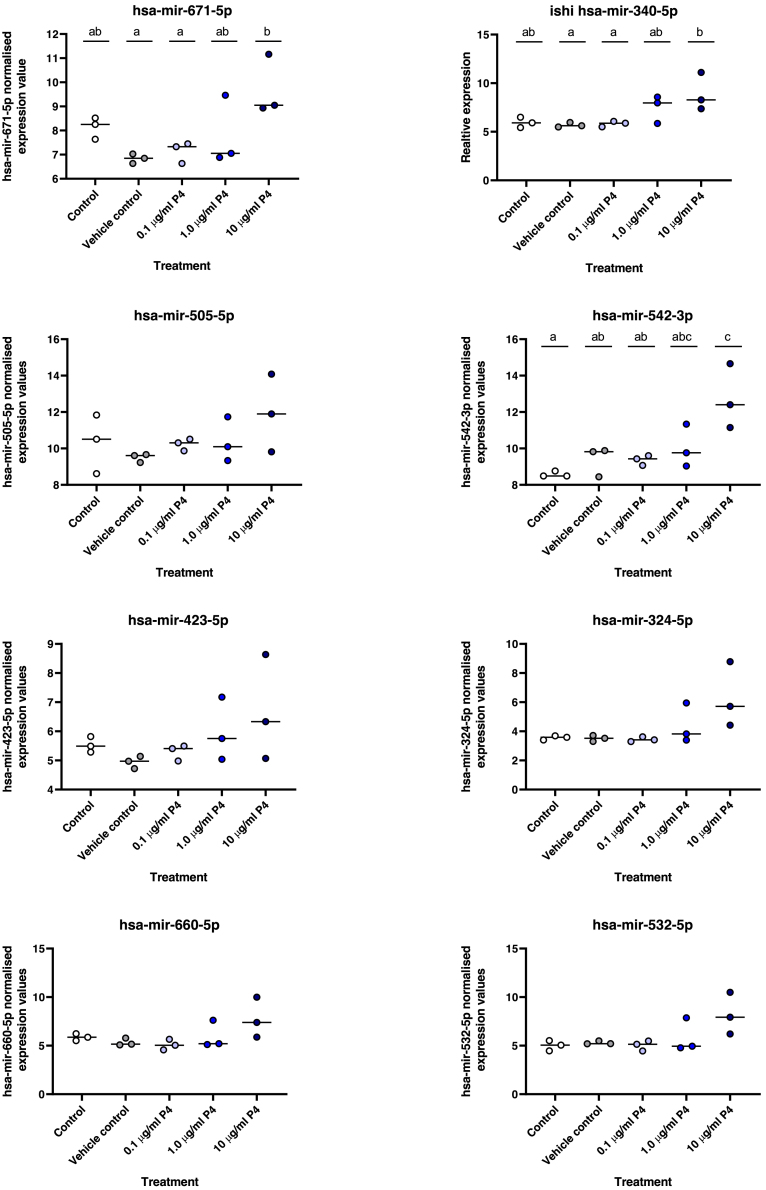

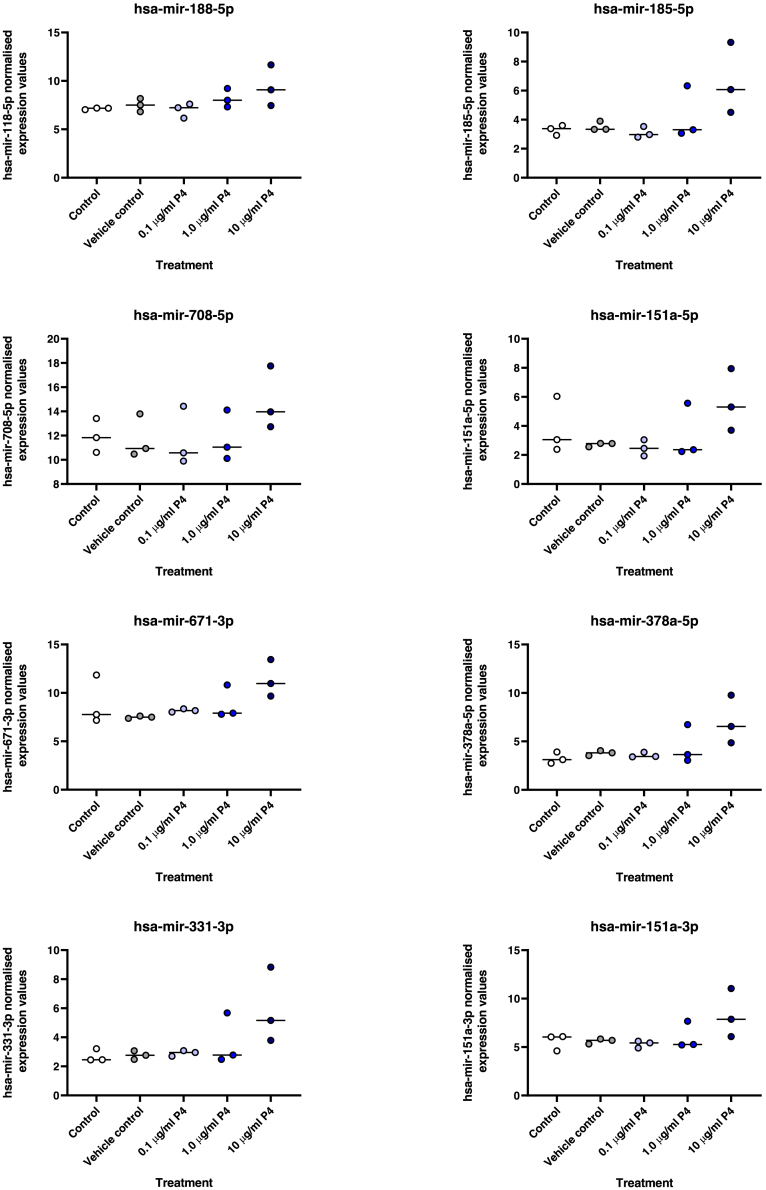
Expression of selected miRNAs in human endometrial epithelial (Ishikawa) cells Cells were cultured for 24 h with: (1) Control (open circle), (2) Vehicle Control (10% EtOH: gray circle), (3) 0.1ug/ml P4 (light blue circle), (4) 1.0 μg/ml P4 (royal blue circle), or (5) 10 μg/ml P4 (dark blue circle: n = 3 biological replicates). Expression values of miRNAs were determined using LNA Sybr Green method and the relative expression values following normalization. Differences in expression were determined relative to vehicle control using an ANOVA and considered statistically different when p< 0.05. Differences between groups are denoted by difference in letters (a, b, c, d).

Relative expression levels of miR-340-5p, -542-3p, and -671-5p all increased following treatment with progesterone (10 μg/mL) for 24 h (Figure 1) compared to control(s). No treatment effect was observed for the other miRNAs (p>0.05). TargetScan predicted miR-340-5p has binding sites in 6,558 human transcripts. Of these predicted targets 68% (4,434) have functional assignments to ontologies/pathways in the interactome database, i.e., have an assigned function. For miR-542-3p, 4,748 targets were predicted using TargetScan and 2041 have assigned functions in the reactome database. In the case of miR-671-5p 2820 of 4,224 predicted targets have a function in the reactome database.

**Figure 2:**
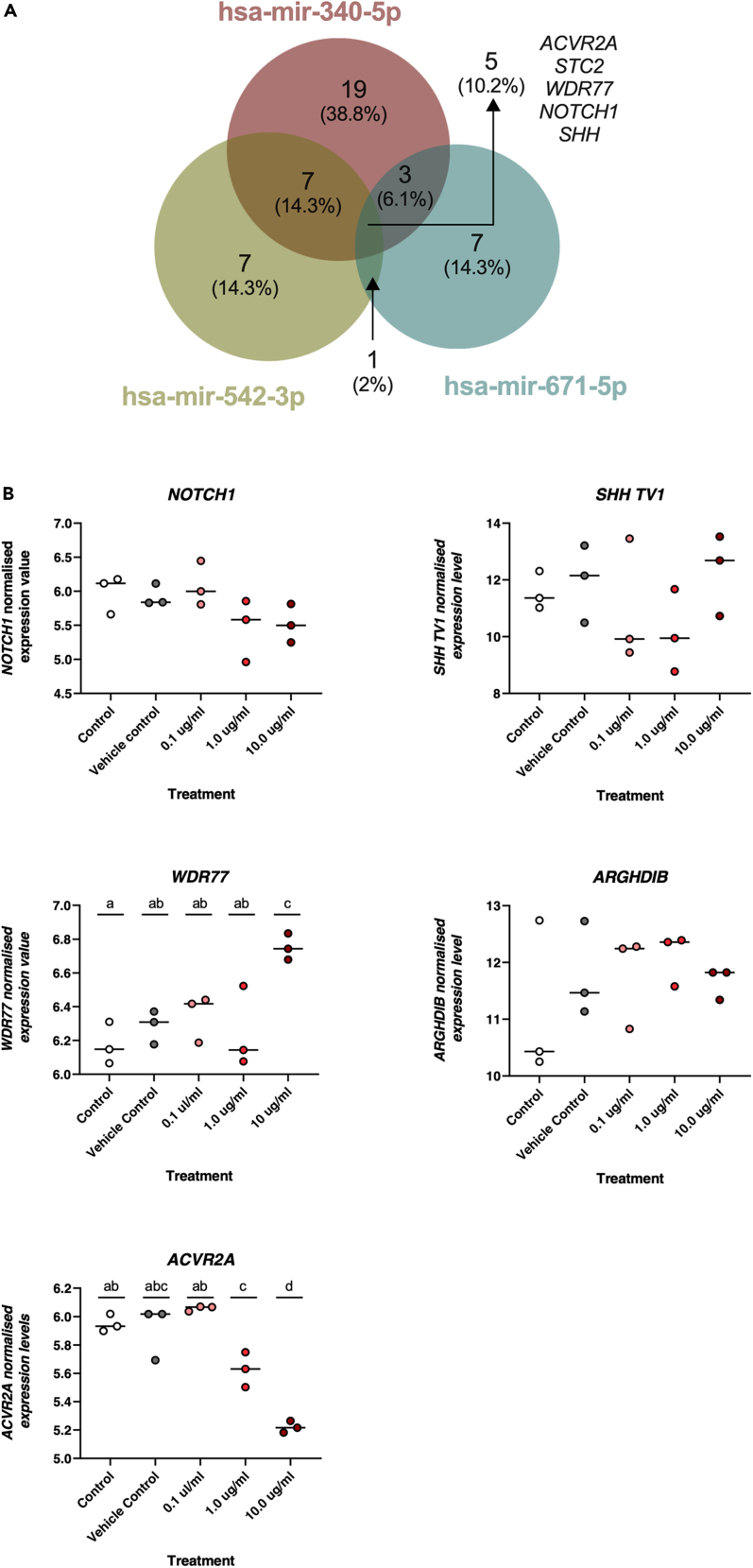
Progesterone regulated microRNA targets (A)Venn diagram representing overlap in predicted mRNA targets of the three miRNAs altered following 24 h treatment of endometrial epithelial cells with P4 in vitro. These were associated with the Gene Ontology Biological Process of Reproductive Biology. (B) RT-qPCR analysis of predicted targets of all three P4-regulated miRNAs that are associated with the Gene Ontology Biological Process of Reproductive Function. Expression was determined in Ishikawa cells treated with control (open circles), vehicle control (gray circles), 0.1 μg/mL (pale red circle), 1.0 μg/mL (medium red circle), or 10.0 μg/mL (dark red circle) of P4 for 24 h*in vitro* (n = 3 independent cultures). Differences in expression compared were determined using ANOVA with differences depicted by different subscripts (a, b, c, d) when p < 0.05.

In total, 34 predicted targets of mir-340-5p, 20 predicted targets of mir-542-3p, and 16 predicted targets of mir-671-5p were associated with the GO term ‘reproductive function’ and were thus selected for further investigation. There was considerable overlap in the predicted ‘reproductive function’ targets across these three microRNA mature sequences where mir-542-3p and mir-671-5p had seven unique target predictions each (Figure 2A), conversely 19 were predicted to be targets of miR-340-5p alone. Common to all three P4-dependent miRNAs (i.e., mir-340-5p, mir-542-3p and mir-671-5p) were five ‘reproductive function’ predicted targets (Activin A Receptor, Type IIA [*ACVR2A*], Stanniocalcin-Related Protein [*STC2*], WD Repeat-Containing Protein 77 [*WDR77*], Notch Receptor 1 [*NOTCH1*], and Sonic Hedgehog Signaling Molecule [*SHH*]. On examining these five common mRNA targets (Table 1) we found there was no effect of P4 on expression of *ARGHDIB*, *NOTCH1*, or *SHH* between any groups (Figure 2B:(p > 0.05)). Expression of *WDR77* increased in cells treated with the highest dose of P4 compared to controls (p < 0.05), whereas expression of *ACVR2A* expression decreased in cells treated with 1.0 and 10.0 μg/ml of P4 compared to control(s) (p < 0.05).

**Table 1:** Primers used for q-RT-PCR analysis of P4 modified transcripts related to reproductive function

Gene abbreviation	Accession No	Forward primer sequence	Primer length	Amplicon length
*ACVR2A*	NM_001278579.1	Forward: TCCGAGGAAGACCCAGGGAA	20	133
		Reverse: GTATAGCACCTGAAGAACAGGAGA	24	
*STC2*	NM_003714.2	Forward: CAGTTGCAGCGGGAATGCTA	20	123
		Reverse: GAGGTCCACGTAGGGTTCGT	20	
*WDR77*	NM_001317062.1	Forward: CCCAACGAAGGCTTCTGCTC	20	111
		Reverse: TTCAACAGCACCTGAATCGGA	21	
*NOTCH1*	NM_017617.5	Forward: GGCCACCCCTCCTAGTTTGG	20	148
		Reverse: CCTCACTGGCATGACACACAA	21	
*SHH*	NM_000193.4	Forward: CACCCCCAATTACAACCCCG	20	80
		Reverse: TACACCTCTGAGTCATCAGCCT	22	

Gene name, accession number, forward primer sequence, length, reverse primer sequence, length, and amplicon length used for q-RT-PCR analysis of P4-regualted transcripts associated with reproduction function that are also predicted targets of P4-regualted miRNAs.

### Coordinate changes in mRNAs in endometrial epithelial cells treated with progesterone

**Figure 3:**
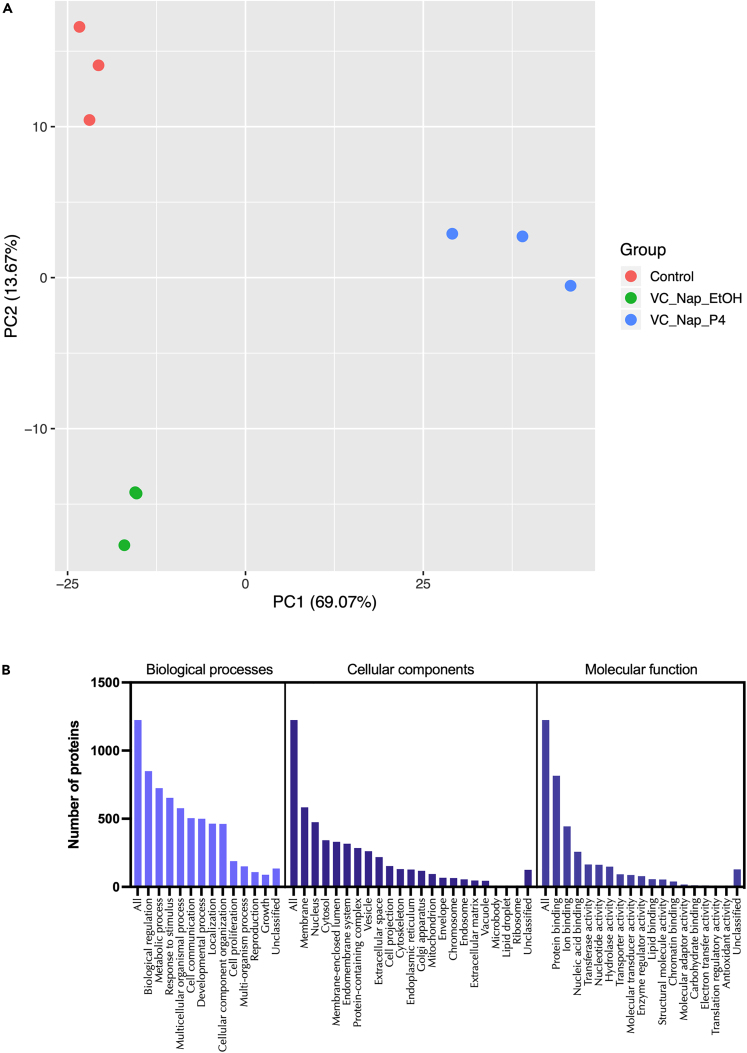
RNA-Seq analysis of Ishikawa cells treated with P4 in vitro (A) Principal component analysis depicting the overall transcriptional differences between cells via RNA sequencing of Ishikawa cells treated with (1) Control (orange circle), (2) Vehicle control (green circle), and (3) 10 μg/ml P4 (blue circle) for 24 h. Controls clearly separate on the left-hand side whereas P4 treatment clearly cluster together on the right-hand side of the graph (n = 3 biological replicates per group). (B) Bar chart of Biological Processes (red), Cellular Component (blue), and Molecular Function (green) associated with P4 modified transcripts.

Next, we sought to identify coordinate changes in mRNA expression occurring with the changes in miRNA expression at the highest concentration of P4. Principal Component Analysis (PCA) of RNA sequencing data revealed clear separation between controls and P4 treated cells (Figure 3A). Following data quality control step and a Benjamini–Hochberg FDR correction, differentially expressed protein coding genes (DEGs) were determined. In total, 6,367 DEGs were identified, 1,238 of which had a log2 fold change of greater than 1 (Table S1).

**Figure 4:**
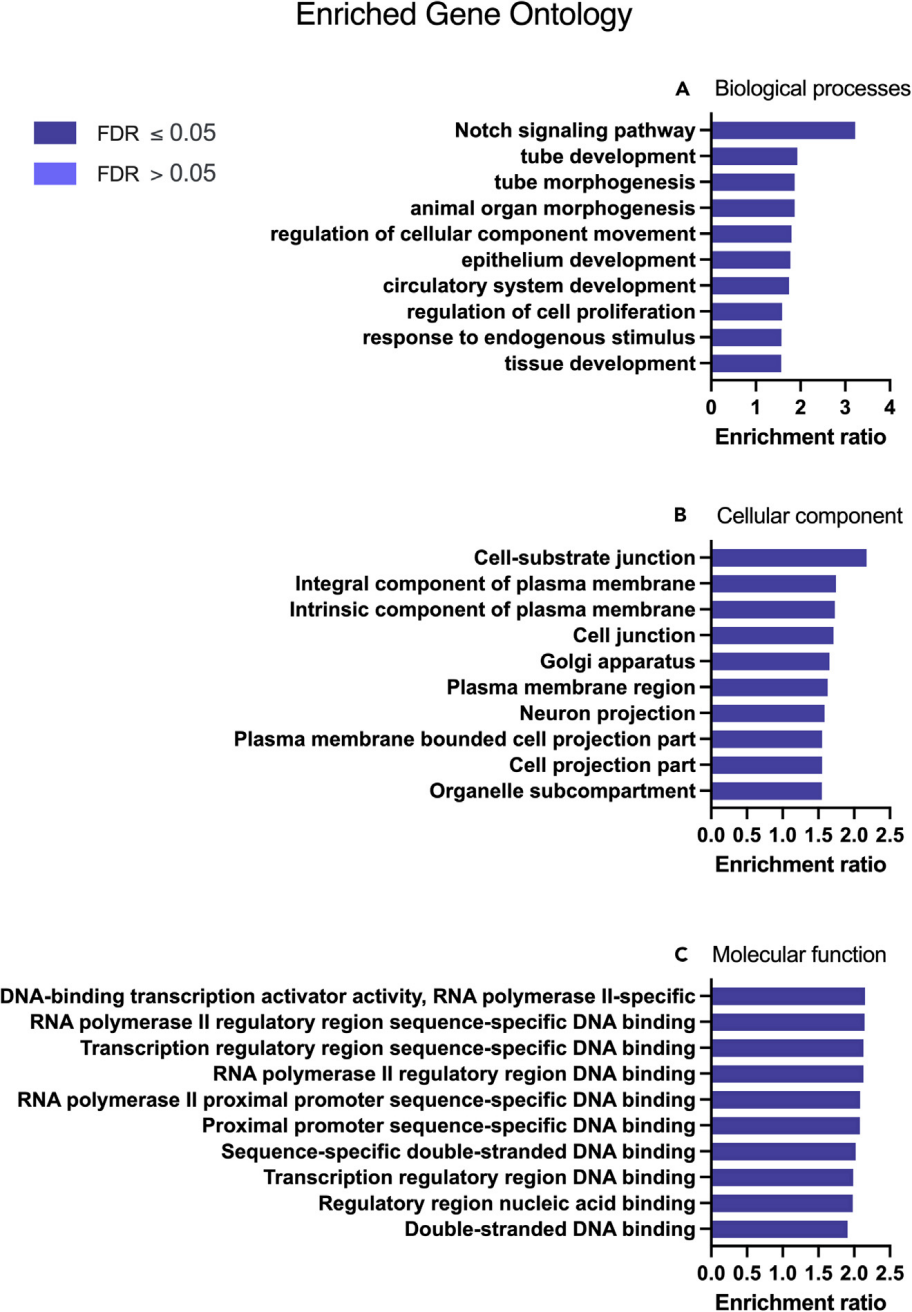
Enriched Gene Ontology associated with P4 modified transcripts (A) Biological processes, (B) Cellular component, and (C) Molecular functions associated with P4 modified transcripts determined following RNA sequencing of human endometrial epithelial cells (Ishikawa cells) treated with 10 μg/ml P4 for 24 h. These are significantly enriched i.e. more transcripts associated with these ontologies than one would expect by chance (FDR <0.05).

**Figure 5:**
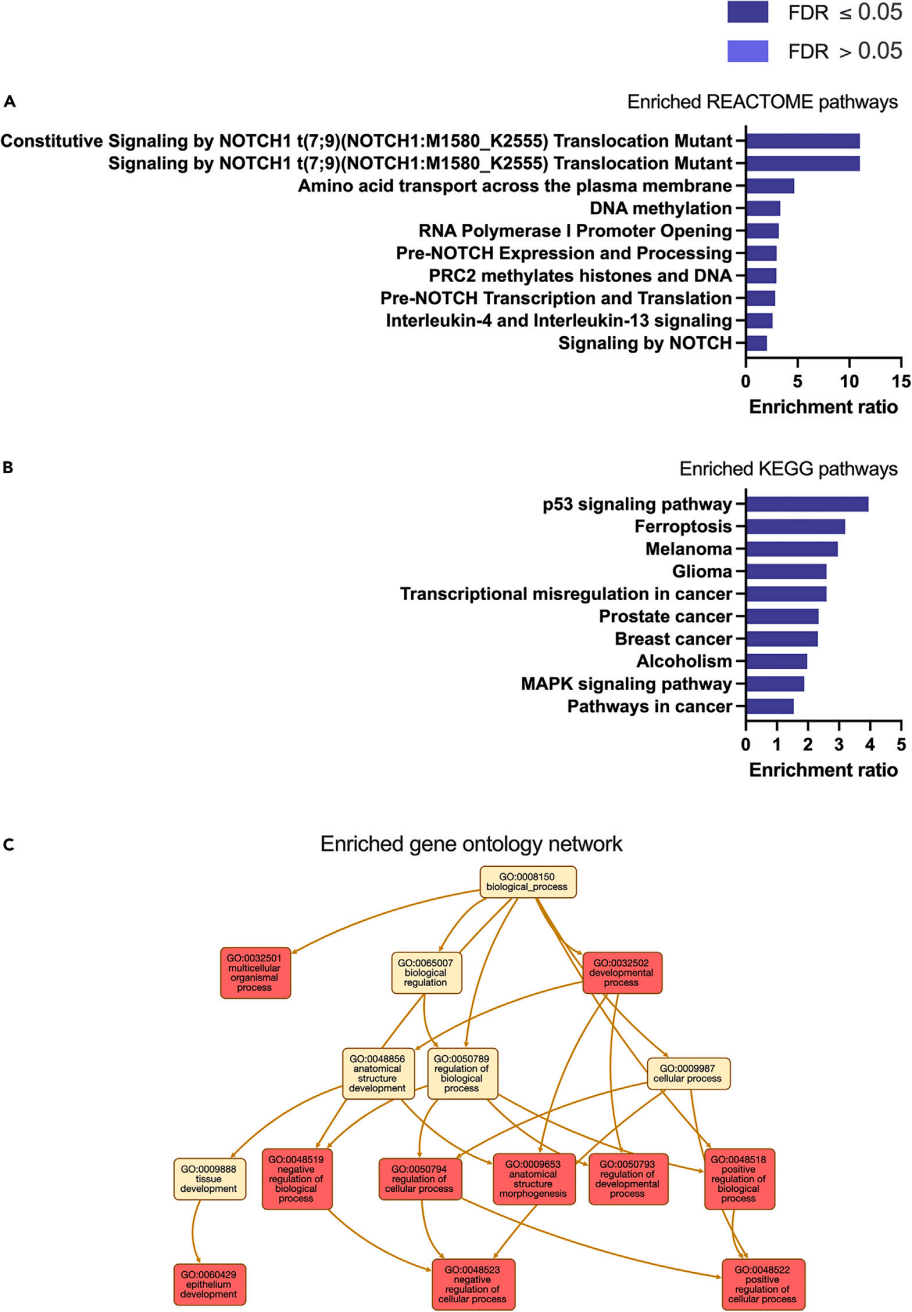
Enriched pathways and networks associated with P4-modified transcripts (A) REACTOME pathways, (B) KEGG pathways, and (C) ontology network, associated with P4 modified transcripts determined following RNA sequencing of human endometrial epithelial cells (Ishikawa cells) treated with 10 μg/ml P4 for 24 h. These are significantly enriched i.e. more transcripts associated with these pathways than one would expect by chance (FDR <0.05).

P4 altered the expression of transcripts in the categories of: biological regulation, metabolic processes, localized to the membrane and to the nucleus, and molecular functions of protein, ion, and nucleic acid binding more than was expected by chance (Figure 3B). Biological processes enriched in these P4 treated cells included Notch signaling pathway, tube development, tube morphogenesis, and epithelium development (Figure 4A: Table S2). Cellular components were enriched in ‘cell-substrate junction’, and ‘integral component of plasma membrane’ (Figure 4B: Table S3), whereas molecular functions influenced by P4 treatment included DNA-binding transcription activator activity, RNA polymerase II regulatory region sequence-specific interactions (Figure 4C: Table S4). The signaling pathways that were enriched involved signaling by NOTCH1, amino acid transport and DNA methylation (Figure 5A: Table S5) whereas enriched KEGG pathways were involved in p53 signaling, ferroptosis, melanoma, and glioma (Figure 5B: Table S6). The gene ontology enrichment network used a total of 22,194 genes with 1,089 seeds in the selected network. A total of 844 seeds were identified in the expanded sub-network and they produced an enriched biological process network including those involved in developmental processes, epithelial development, and both positive and negative regulation of cellular processes (Figure 5C).

**Figure 6:**
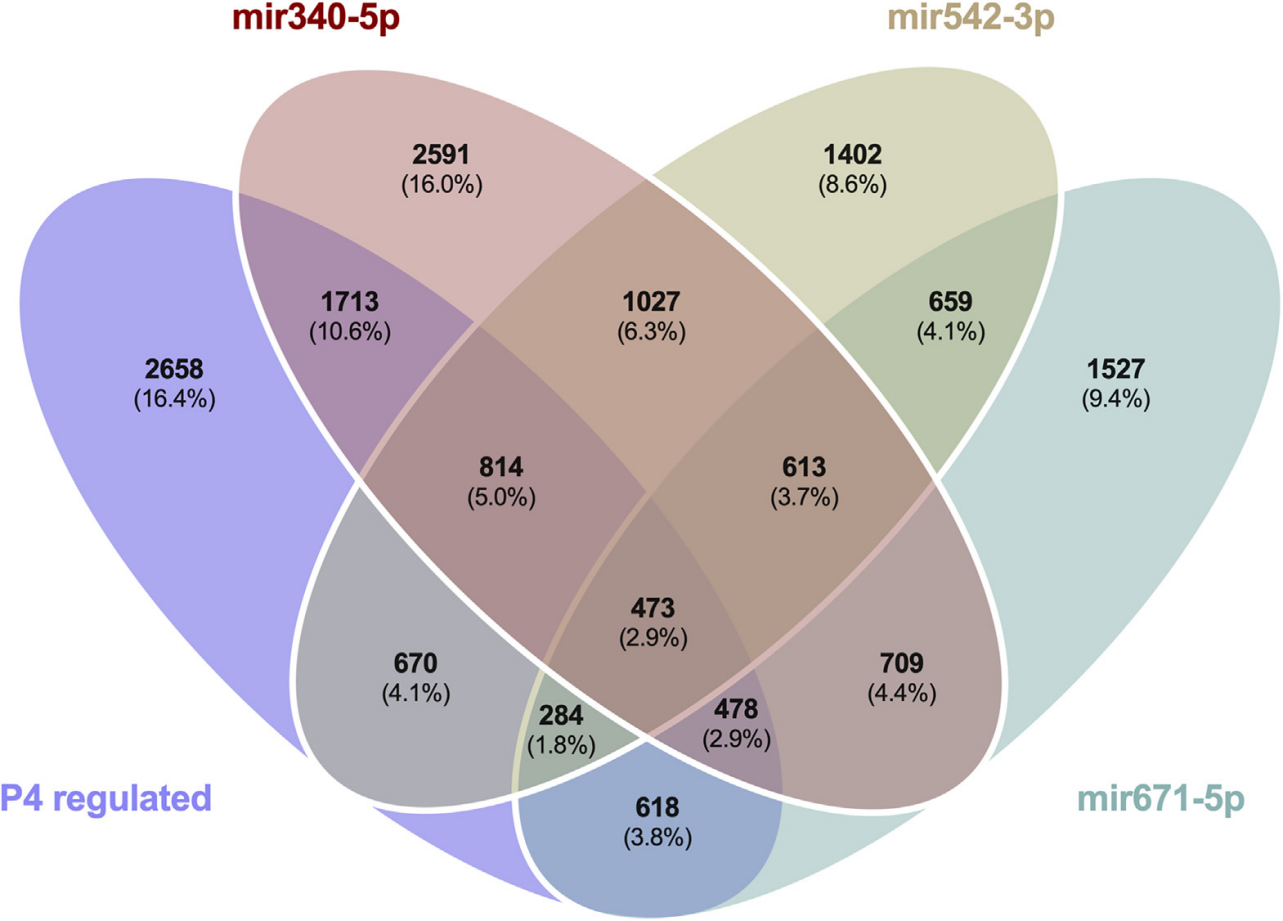
Overlap of predicted miRNA targets and P4 altered transcripts Venn diagram analysis demonstrating the overlap in predicted targets (from miRBase) of the 3 miRNAs modified by treatment of Ishikawa cells with P4 for 24 h*in vitro* as well as those mRNAs that were identified as altered in the same cells following P4 treatment for 24 h compared to vehicle controls.

**Figure 7:**
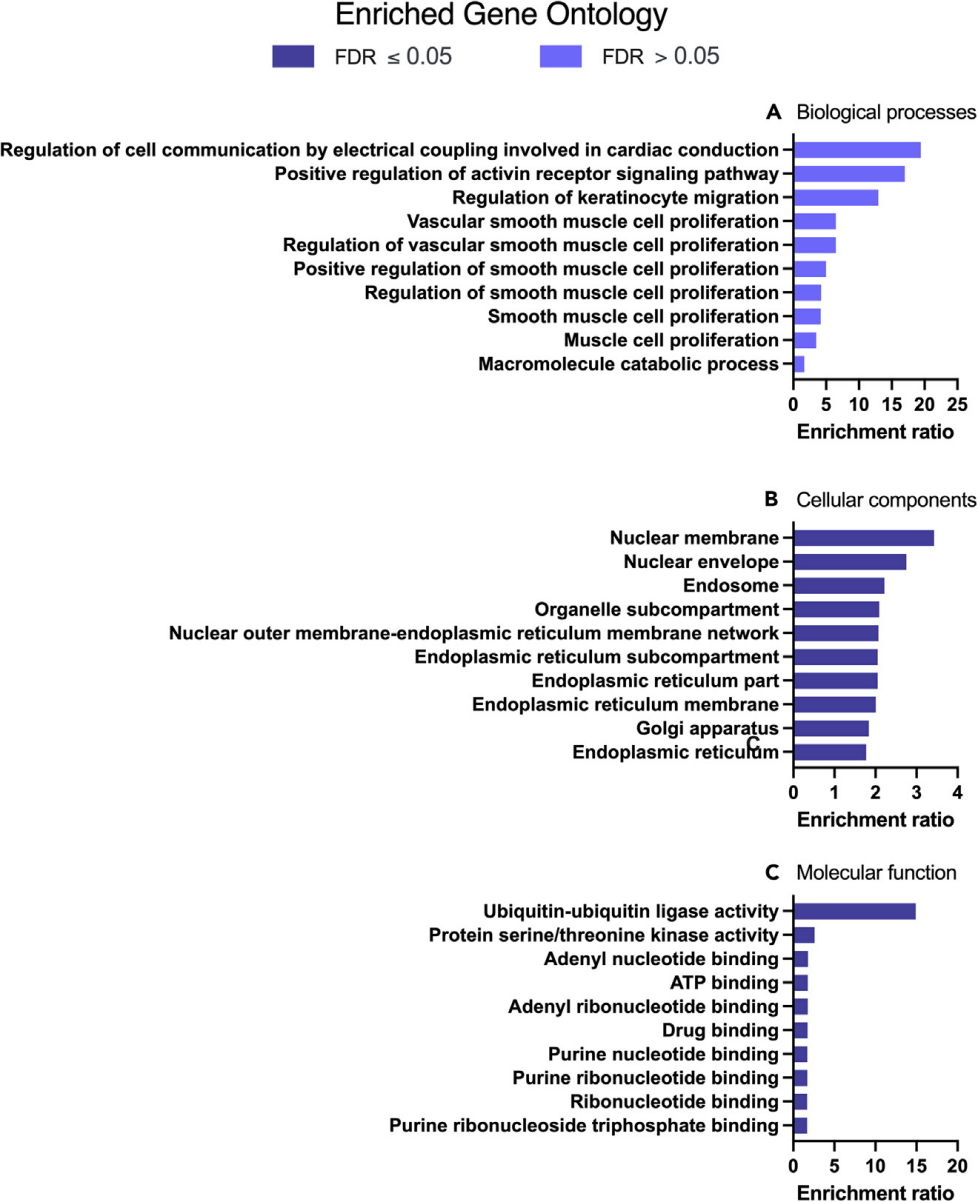
Enriched Gene Ontologies for P4-modified transcripts and miRNA targets (A) Biological processes, (B) Cellular component, and (C) Molecular functions associated with 473 P4 modified transcripts that are also predicted targets of the 3 miRNAs modified in human endometrial epithelial cells (Ishikawa cells) treated with 10 μg/ml P4 for 24 h. These are significantly enriched i.e. more transcripts associated with these ontologies than one would expect by chance (FDR <0.05).

**Figure 8:**
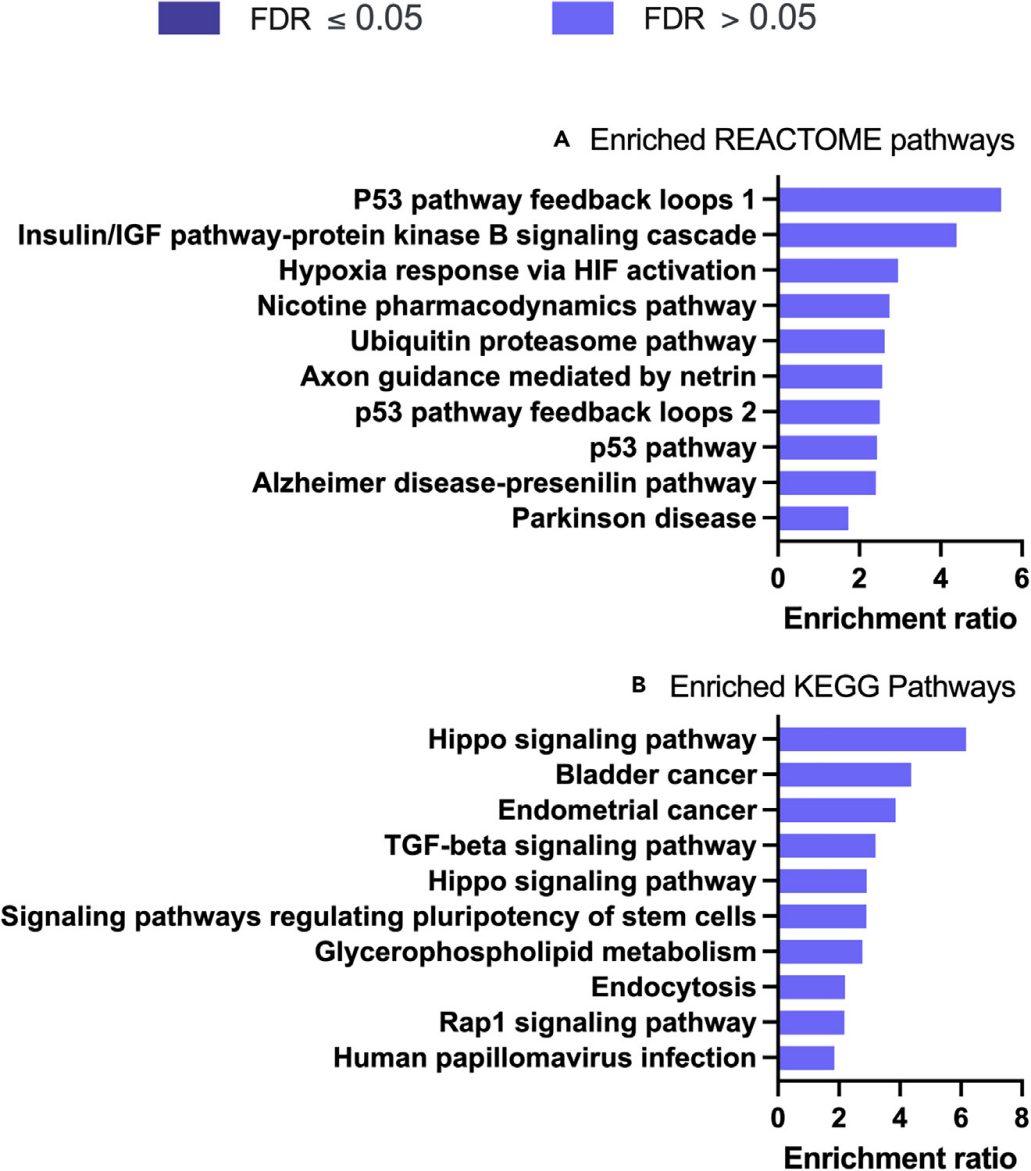
Enriched pathways for P4-modified transcripts and miRNA targets (A) REACTOME pathways, and (B) KEGG pathways associated with 473 P4 modified transcripts that are also predicted targets of the 3 miRNAs modified in human endometrial epithelial cells (Ishikawa cells) treated with 10 μg/ml P4 for 24 h. These are significantly enriched, i.e., more transcripts associated with these ontologies than one would expect by chance when FDR <0.05.

We then investigated the overlap in the P4-dependent expression profiles and the predicted targets from TargetScan of the three P4-regulated miRNAs (Figure 6). Of the predicted targets of miR-340-5p: 1713 transcripts were also changed in P4 treated cells; miR-542-3p: 670 transcripts, and miR-671-5p: 618 transcripts indicating distinct P4-miRNA transcriptomic signatures. Complete details of transcripts are provided in Table S7. A cohort of 473 transcripts that are predicted targets of all three P4-regulated miRNAs exhibit P4-responsive expression in the same cells. No significantly enriched biological processes or KEGG/panther pathways were identified (Figure 7A; Table S8 and Figure 8; Tables S11 and S12). However, significant enrichment for cellular components including those involved in the nuclear membrane, nuclear envelope, and endosome (Figure 7B: Table S9) were observed. Molecular functions of ubiquitin-ubiquitin ligase activity, protein serine/threonine kinase activity, and adenyl nucleotide activity were also significantly enriched (Figure 7C: Table S10).

### *In vitro* targets of miR-340-5p in endometrial epithelial cells

**Figure 9:**
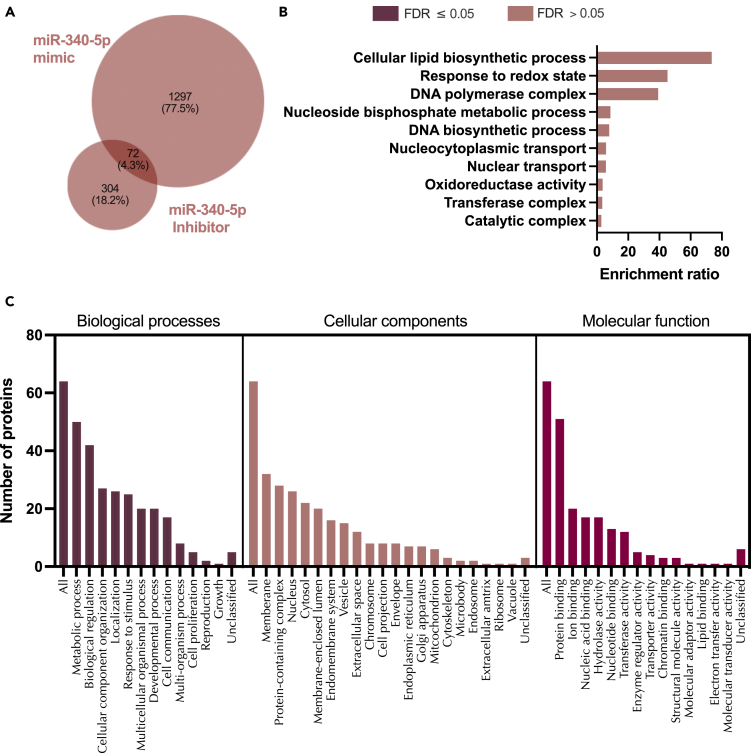
Proteins altered in Ishikawa cells following treatment with miR-340-5p mimic and inhibitor (A) Venn diagram depicting total number of significantly differentially expressed proteins (p<0.05) following transfection of Ishikawa cells (n = 3 biological replicates) with miR-340-5p mimic (RHS) and inhibitor (LHS). (B) Enriched KEGG pathways associated with miR-340-5p mimic and inhibition regulated proteins. (C) WebGestalt overrepresentation analysis of biological process, cellular component and molecular function categories for identified significantly differentially expressed proteins (p<0.05) in response to miR-340-5p mimic and inhibition (total of 72).

Excluding off target effects, treatment of endometrial epithelial cells with mir-340-5p mimic altered 1,369 proteins as compared to controls (p < 0.05; Figure S1; Table S22). These were significantly enriched in pathways involved in oxidation-reduction function and metabolism pathways (Figure S1). In contrast, inhibition of mir-340-5p changed expression of 376 proteins (p < 0.05; Figure S2; Table S23) including those involved in tRNA and ncRNA processing as well as nuclear transport (Figure S2). A cohort of 72 proteins was altered in endometrial epithelial cells following treatment with both mimic and inhibitor of mir-340-5p (Figure 9; Table S13).

**Figure 10:**
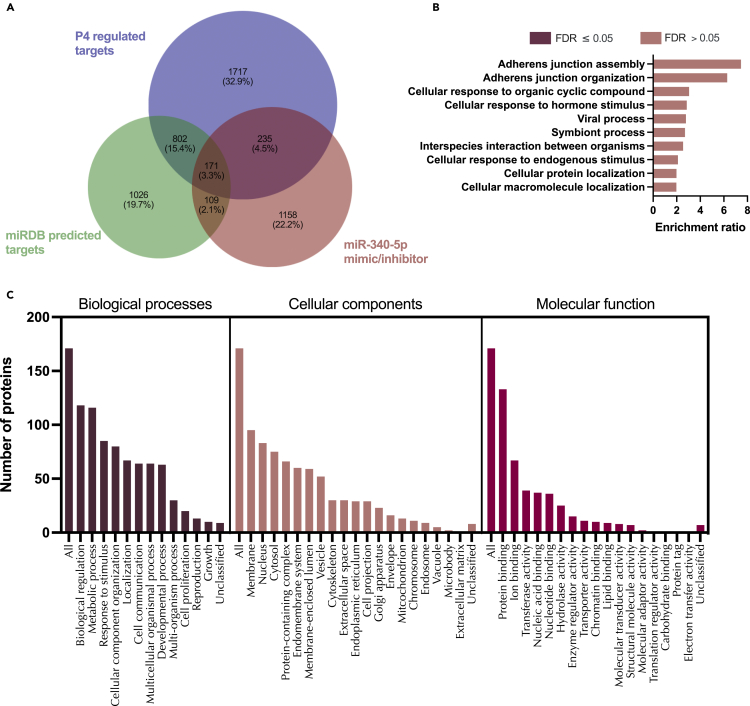
Proteins altered in Ishikawa cells following treatment with miR-340-5p mimic and inhibitor compared to mirDB predicted targets and P4 regulated mRNAs (A) Venn diagram depicting total number of significantly differentially expressed proteins (p<0.05) following transfection of Ishikawa cells (n = 3 biological replicates) with miR-340-5p mimic or inhibitor vs non-targeting controls and mirDB predicted targets, or P4-regulated transcripts. (B) Enriched KEGG pathways associated with miR-340-5p mimic and inhibition regulated proteins, P4-regulated mRNAs and predicted target overlap. (C) WebGestalt overrepresentation analysis of biological process, cellular component and molecular function categories for identified significantly differentially expressed proteins (p<0.05) in response to miR-340-5p mimic and inhibition, predicted targets, and P4-regulated mRNAs overlap.

We then sought to determine how many of these *in vitro* identified targets of mir-340-5p were predicted in mirDB. A total of 280 proteins were identified between predicted (mirDB) and confirmed (*in vitro*) targets (Figure S3). Only 6.9% of total predicted targets were changed in endometrial cells treated with mir-340-5p mimic whereas 1.3% were altered following inhibition of mir-340-5p (Figure S3: Table S24). These proteins were overrepresented in pathways involving the nuclear pore, mRNA-3′-UTR binding, and cadherin binding (Figure S3). Finally, we compared our *in vitro* mimic/inhibitor list of proteins, mirDB predicted targets, and those mRNAs altered by P4 in endometrial epithelial cells. In total, 171 proteins predicted to be targets by mirDB were altered *in vitro* by treatment with miR-340-5p mimic or inhibitor and were also altered by treatment of endometrial epithelial cells with P4. These proteins were involved in biological processes involving developmental processes, cell proliferation, and reproduction amongst others and are likely key components of P4 regulation of the endometrial epithelium (Figure 10, Table S14).

**Figure 11:**
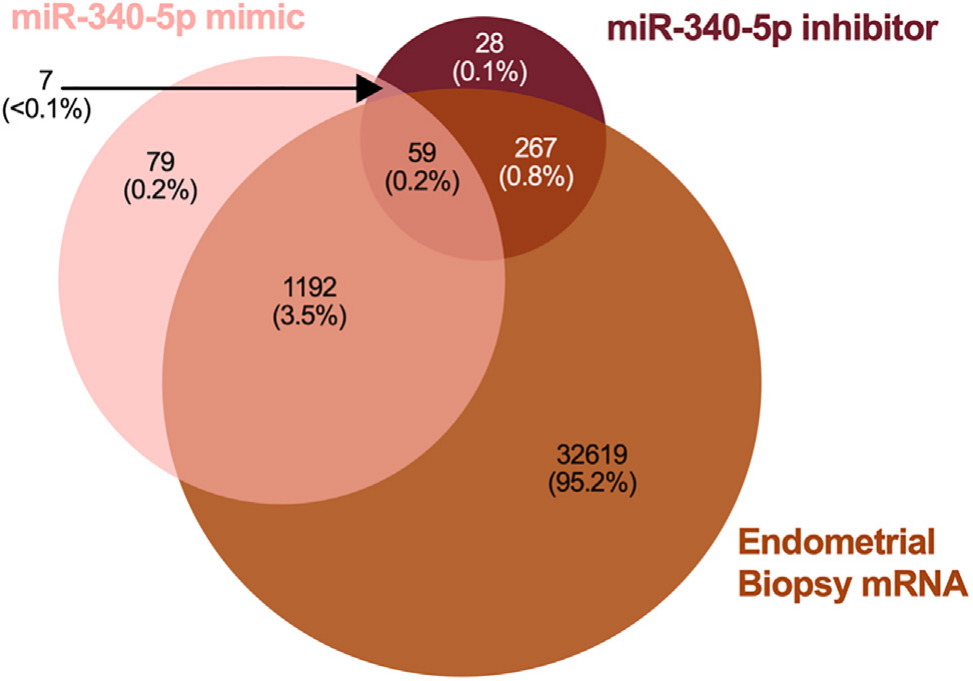
Comparison of miR-340-5p-regulated proteins to endometrial biopsy transcript expression Venn diagram showing overlap in proteins altered in abundance following transfection of Ishikawa cells (n = 3 biological replicates) with miR-340-5p mimic or inhibitor compared to RNA-Seq data from human endometrial biopsies (n = 36 biological replicates).

We wished to investigate whether the mRNA counterparts of proteins changed in response to miRNA mimics and inhibitors were present in RNA-Seq data from human endometrial biopsy samples.^21^ Of the proteins altered by miR-340-5p mimic, 94% of their corresponding mRNAs were detected in patient samples. Furthermore, only 13 of 1251 mRNAs were absent from 1 or more of n = 36 samples. Ninety percent of proteins affected by inhibition of miR-340-5p were identified in RNA-Seq data, with only 5 of 326 mRNAs not present in all of the 36 patient samples. There were 59 of each of these sets of proteins which were changed by both mimic and inhibitor whose mRNA was found in patient samples (Figure 11, Tables S15A–S15D).

### *In vitro* targets of miR-542-3p in endometrial epithelial cells

**Figure 12:**
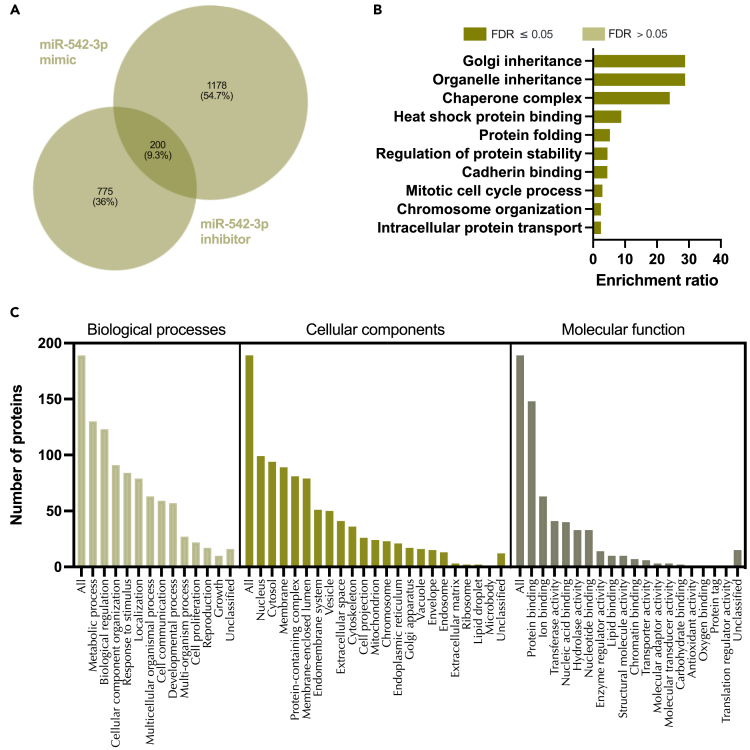
Proteins altered in Ishikawa cells following treatment with miR-542-3p mimic and inhibitor (A) Venn diagram depicting total number of significantly differentially expressed proteins (p<0.05) following transfection of Ishikawa cells (n = 3 biological replicates) with miR-542-3p mimic (RHS) and inhibitor (LHS). (B) Enriched KEGG pathways associated with miR-542-3p mimic and inhibition regulated proteins. (C) WebGestalt overrepresentation analysis of biological process, cellular component and molecular function categories for identified significantly differentially expressed proteins (p<0.05) in response to miR-542-3p mimic and inhibition (total of 200).

Exclusion of off target effects identified 1,378 proteins altered by treatment of endometrial epithelial cells with miR-542-3p mimic (Figure S4; Table S25). These targets were overrepresented in pathways involved in ribonucleoprotein complex biogenesis and various metabolic processes (Figure S4). Inhibition of miR-542-3p altered 975 proteins including those implicated in pathways related to RNA splicing, mRNA processing, and cellular protein localization (Figure S5, Table S26). A core of 200 proteins was differentially abundant in response to treatment with both miR-542-3p mimic and inhibitor treatment (Figure 12, Table S16). These proteins were enriched pathways involving organelle and Golgi inheritance as well as protein folding (Figure 12).

**Figure 13:**
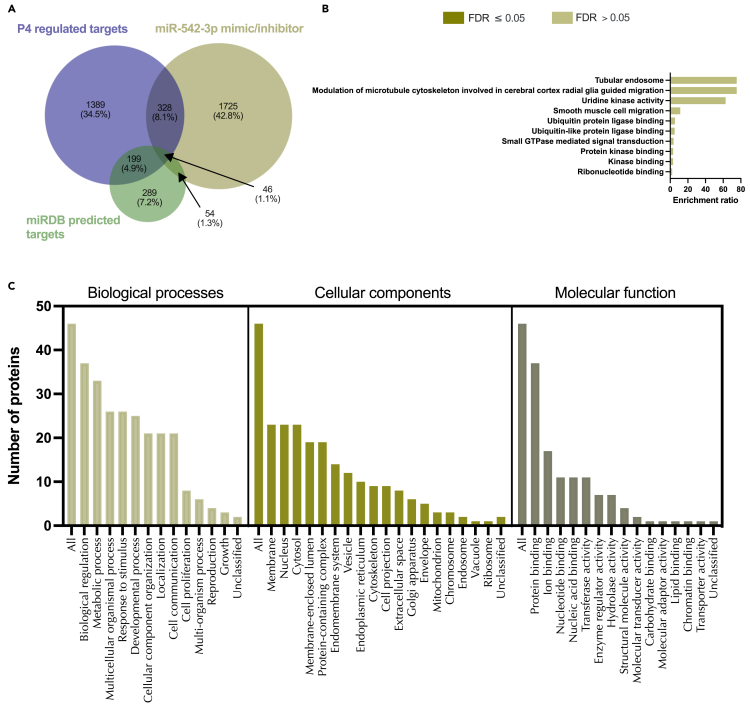
Proteins altered in Ishikawa cells following treatment with miR-542-3p mimic and inhibitor compared to mirDB predicted targets and P4 regulated mRNAs (A) Venn diagram depicting total number of significantly differentially expressed proteins (p<0.05) following transfection of Ishikawa cells (n = 3 biological replicates) with miR-542-3p mimic or inhibitor vs non-targeting controls and mirDB predicted targets, or P4-regulated transcripts. (B) Enriched KEGG pathways associated with miR-542-3p mimic and inhibition regulated proteins, P4-regulated mRNAs and predicted target overlap. (C) WebGestalt overrepresentation analysis of biological process, cellular component and molecular function categories for identified significantly differentially expressed proteins (p<0.05) in response to miR-542-3p mimic and inhibition, predicted targets, and P4-regulated mRNAs overlap.

On comparing these *in vitro* identified targets of miR-542-3p to those predicted by mirDB we identified 100 protein targets in common (Figure S6, Table S27). These were then compared to P4-regulated mRNAs and were assessed for the following criteria: (1) Identified significantly differentially expressed proteins (p<0.05) in response to miR-542-3p mimic and/or inhibitor, (2) mirDB predicted targets of miR-542-3p, and (3) progesterone regulated miRbase targets of miR-542-3p (Figure 13, Table S17). In total, only 46 proteins satisfied all three of our criteria and there are no significantly enriched pathways associated with these 46 proteins; however, they appear to be enriched in thermogenesis (Figure 13).

**Figure 14:**
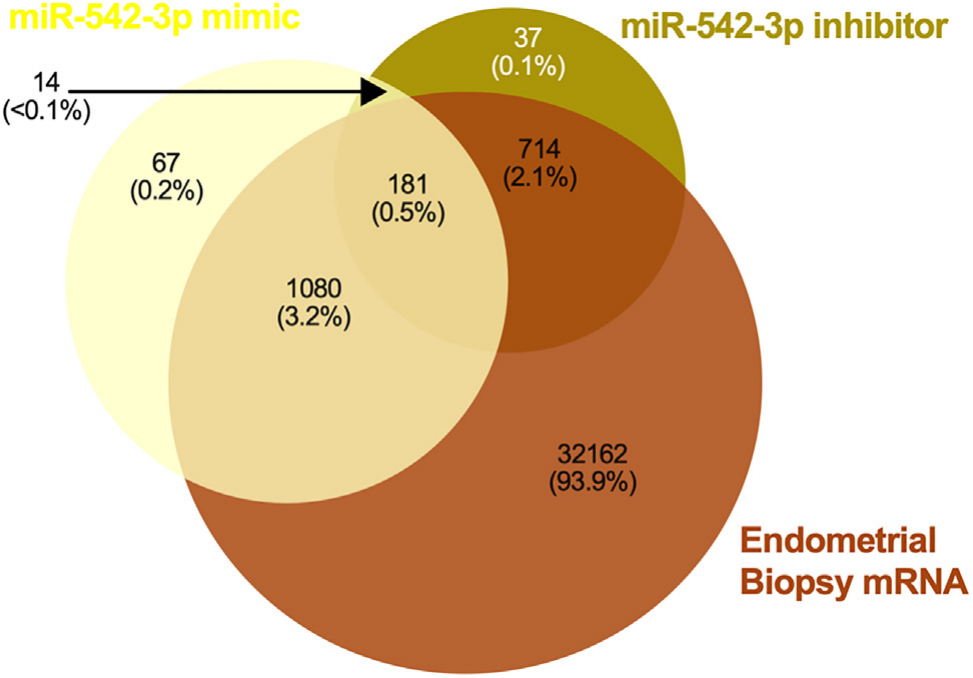
Comparison of miR-542-3p-regulated proteins to endometrial biopsy transcript expression Venn diagram showing overlap in proteins altered in abundance following transfection of Ishikawa cells (n = 3 biological replicates) with miR-542-3p mimic or inhibitor compared to RNA-Seq data from human endometrial biopsies (n = 36 biological replicates).

We carried out a comparison of proteomic data to human endometrial biopsies to determine whether proteins altered by miR-542-3p mimic and/or inhibitor were present in clinical samples.^21^ It was found that 94% of proteins changed in response to miR-542-3p mimic mRNA could be identified in RNA-Seq data. Of these 1,261 proteins, only 11 were absent from 1 or more of n = 36 samples. RNA-Seq data showed presence of mRNA corresponding to 95% of proteins altered in abundance by miR-542-3p inhibitor, with 8 of these 895 mRNAs not recognized in all 36 patients’ endometrial biopsies. A total of 181 proteins common to both mimic and inhibitor altered lists were found in RNA-Seq data (Figure 14, TablesS18A–S18D)

### *In vitro* targets of miR-671-5p in endometrial epithelial cells

**Figure 15:**
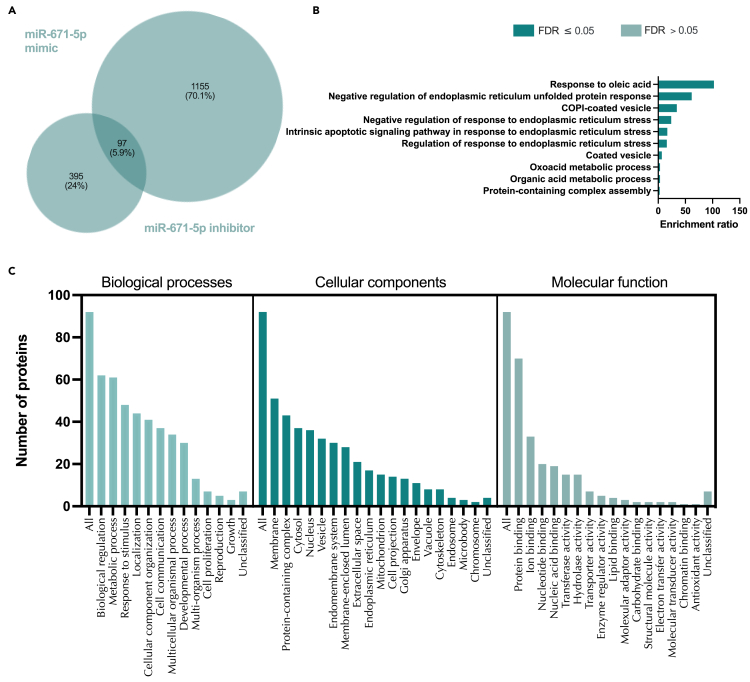
Proteins altered in Ishikawa cells following treatment with miR-671-5p mimic and inhibitor (A) Venn diagram depicting total number of significantly differentially expressed proteins (p<0.05) following transfection of Ishikawa cells (n = 3 biological replicates) with miR-671-5p mimic (RHS) and inhibitor (LHS). (B) Enriched KEGG pathways associated with miR-671-5p mimic and inhibition regulated proteins. (C) WebGestalt overrepresentation analysis of biological process, cellular component and molecular function categories for identified significantly differentially expressed proteins (p<0.05) in response to miR-671-5p mimic and inhibition.

Excluding off target effects, treatment of endometrial epithelial cells with a miR-671-5p mimic, 1,252 proteins were significantly changed (p<0.05) following exclusion of off target effects (Figure S7; Table S13). These proteins were involved in processes involving protein transport and localization (Figure S7). Inhibition of miR-671-5p altered 492 proteins enriched in functions such as rRNA metabolic process, and generation of precursor metabolites and energy (Figure S8; Table S13). The number of proteins that were changed in response to both miR-671-5p mimic and inhibitor was 97, with overrepresented of functions related to response to ER stress (Figure 15; Table S28).

**Figure 16:**
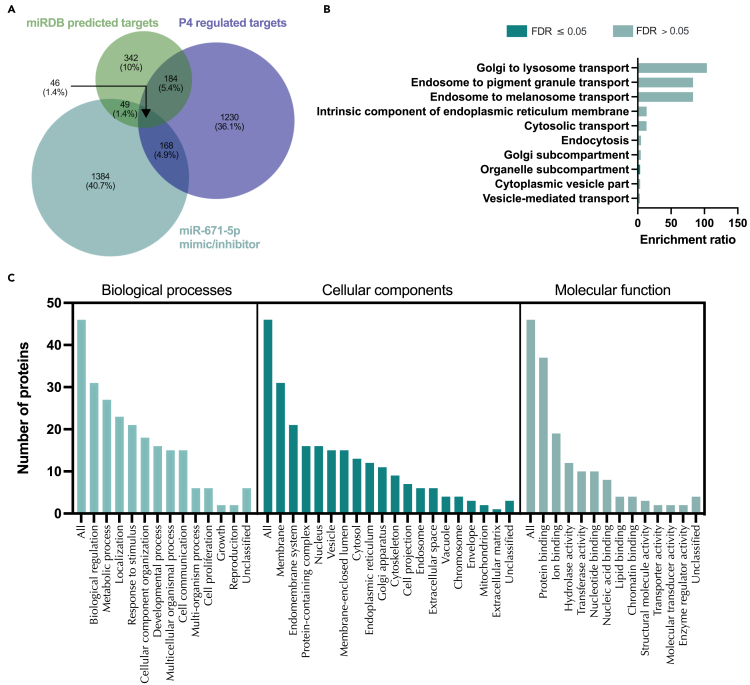
Proteins altered in Ishikawa cells following treatment with miR-671-5p mimic and inhibitor compared to mirDB predicted targets and P4 regulated mRNAs (A) Venn diagram depicting total number of significantly differentially expressed proteins (p<0.05) following transfection of Ishikawa cells (n = 3 biological replicates) with miR-671-5p mimic or inhibitor vs non-targeting controls and mirDB predicted targets, or P4-regulated transcripts. (B) Enriched KEGG pathways associated with miR-671-5p mimic and inhibition regulated proteins, P4-regulated mRNAs and predicted target overlap. (C) WebGestalt overrepresentation analysis of biological process, cellular component and molecular function categories for identified significantly differentially expressed proteins (p<0.05) in response to miR-671-5p mimic and inhibition, predicted targets, and P4-regulated mRNAs overlap.

Comparing the set of proteins that were significantly changed following treatment with miR-671-5p mimic or inhibitor to those predicted to be targets of miR-671-5p, identified 95 proteins in common (Figure S9; Table S30). These proteins were significantly overrepresented (FDR <0.05) for processes involving the Golgi apparatus, cytosolic, intra-cellular, and vesicle-mediated transport (Figure S9). Finally, by comparing proteins that fit each of the following 3 criteria: 1) identified significantly differentially expressed proteins (p < 0.05) in response to miR-671-5p mimic and/or inhibitor, 2) mirDB predicted targets of miR-671-5p, and 3) progesterone regulated miRBase targets of miR-671-5p discovers 46 that are common to all (Figure 16, Table S20). The only significantly enriched category is in their cellular component organelle subcompartment (FDR <0.05). Highly represented processes comprise various transport functions including Golgi to lysosome (Figure 16).

**Figure 17:**
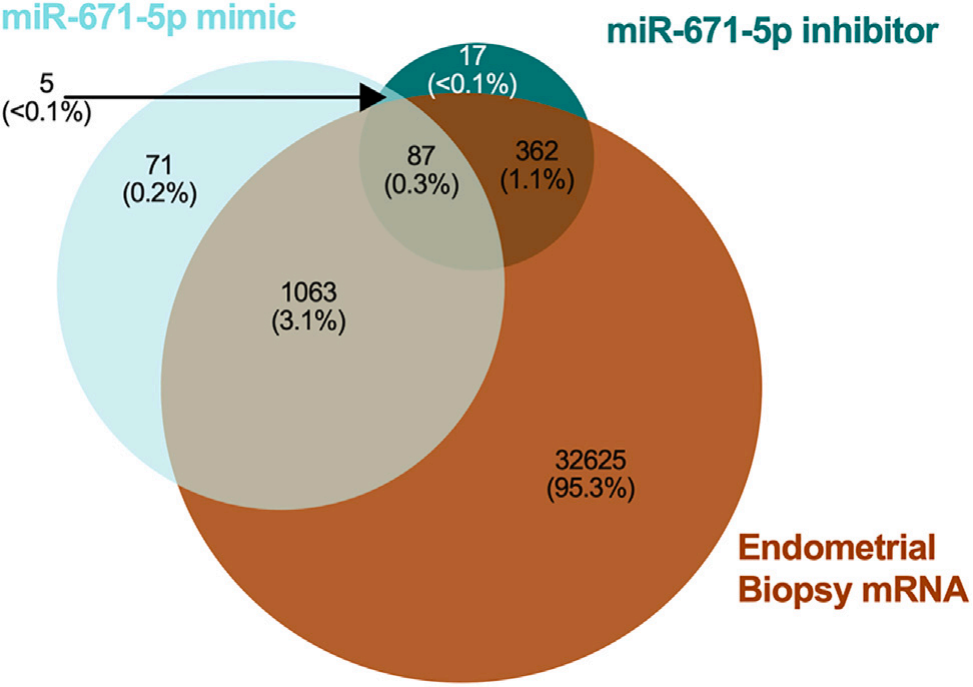
Comparison of miR-671-5p-regulated proteins to endometrial biopsy transcript expression Venn diagram showing overlap in proteins altered in abundance following transfection of Ishikawa cells (n = 3 biological replicates) with miR-671-5p mimic or inhibitor compared to RNA-Seq data from human endometrial biopsies (n = 36 biological replicates).

We examined presence of mRNAs in endometrial biopsies^21^ to find those that correspond to proteins changed in abundance by miR-671-5p mimic and/or inhibitor. This resulted in identification of presence of mRNA for 94% of miR-671-5p mimic affected proteins. Of these 1,150 mRNAs, only 8 were not found in all 36 endometrial samples. Ninety-five percent of proteins changed by the miR-671-5p inhibitor had corresponding mRNAs in patient samples and of those 449, only 8 were absent from 1 or more biopsy. Eighty-seven proteins were altered by both mimic and inhibitor as well as corresponding mRNA detected in endometrial biopsies (Figure 17; Tables S21A–S21D).

### Changes to miRNA profiles in patient samples with different clinical outcomes

**Figure 18:**
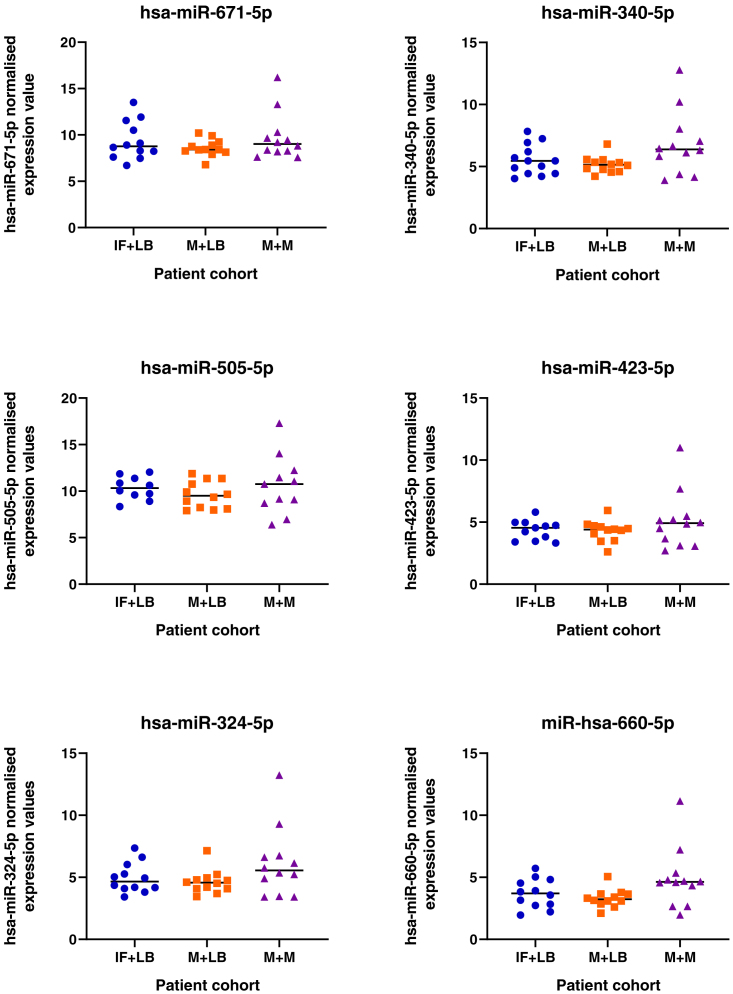

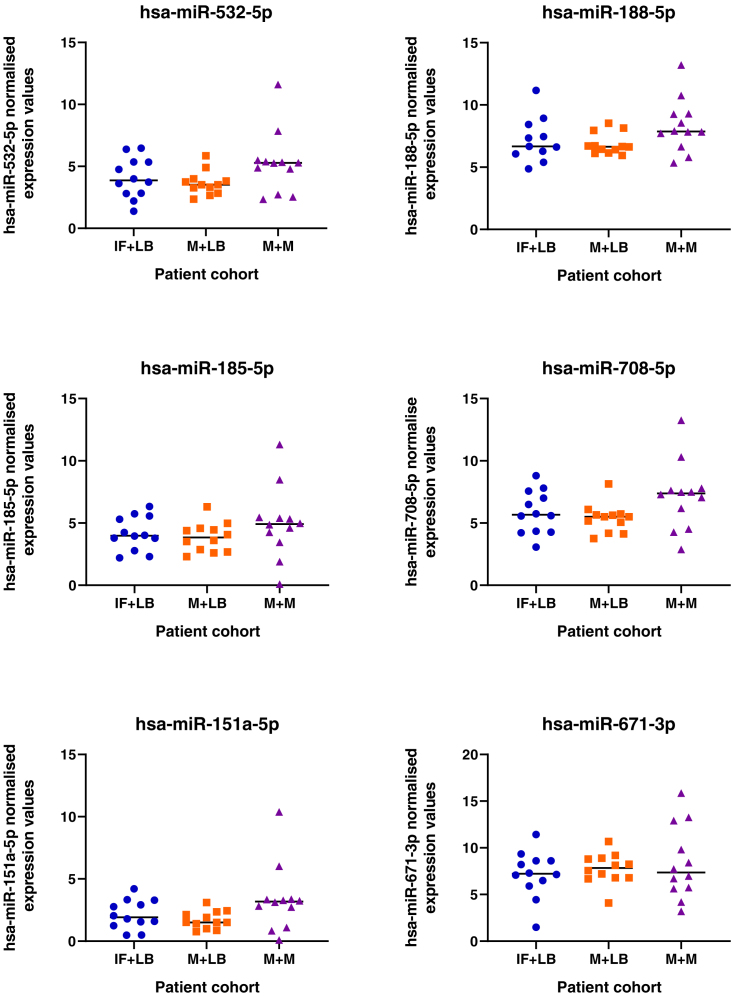

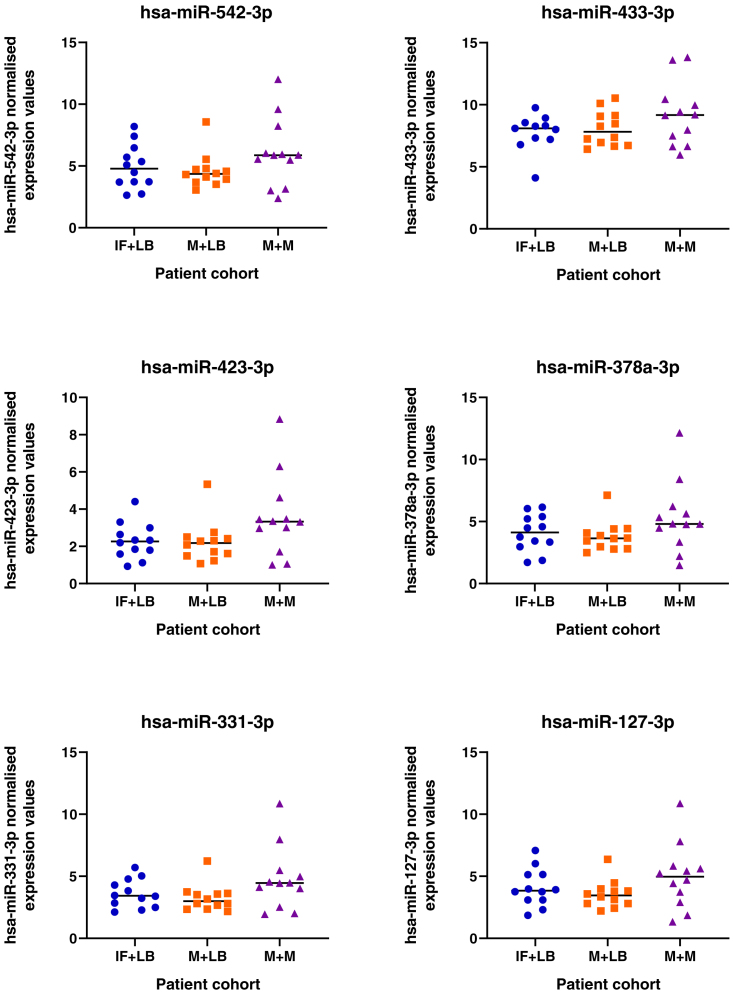

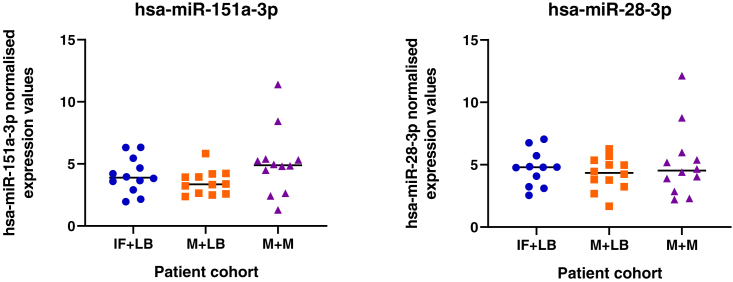
Expression of miRNAs in endometrial biopsy samples Samples were collected from patients during the mid-luteal phase of the menstrual cycle (LH+6-LH+9), from patients (n = 12 per group) who have infertility and a subsequent live birth (IF + LB: blue dots), miscarriage and a subsequent live birth (M + LB: orange square), or miscarriage and a subsequent miscarriage (M + M: purple triangles). Following statistical analysis using an ANOVA, there was an overall effect on mir-340-5p expression (p <0.05) between groups.

Given the pathways these miRNAs regulate *in vitro* we sought to determine if their expression is dysregulated in endometrial biopsies from women who experienced pregnancy loss during the luteal phase of the cycle. We detected expression of all miRNAs in endometrial biopsies taken from patients during the luteal phase of their cycle, irrespective of prior or future pregnancy outcomes (Figure 18). No significant difference between patient cohorts was determined for 19 of the 20 mature seeds from the 13 microRNA families. However, expression of mir-340-5p showed an overall increase in expression in patients who had previously suffered a miscarriage and had a subsequent miscarriage, as compared to those who had infertility or previous miscarriage and subsequently went on to have a life birth outcome.

## Discussion

We tested the hypothesis that a set of miRNAs whose evolutionary appearance coincides with the emergence of placental mammals and were retained in all placental mammal lineages thereafter may play a role in uterine function for successful pregnancy in humans.^18^ The rationale for this is the fact that the endometrium and trophoblast have had to co-evolve regulatory signaling networks and the dysregulation of these networks may contribute to pregnancy loss. P4 signaling during the menstrual cycle is critical to establish receptivity of the endometrium to implantation.^22^ In contrast with the follicular phase during which the expression of estrogen receptors are high, during the P4-dominant luteal phase, P4 suppresses ESR expression and increases and then modifies PGR expression in a spatial and temporal manner.^23^ Rising concentrations of P4 results in halted epithelial proliferation and increased stromal cell proliferation, priming the endometrium for implantation and subsequent decidualization.^24^ Epithelial cells begin to differentiate, and tight junctions and adherens in the luminal epithelium are disrupted.^25^ An important P4 target, is Indian hedgehog (*Ihh),* predominantly expressed in the epithelium and is required for endometrial receptivity. It facilitates communication between epithelial and stromal cells, and knockout of its expression in the endometrium results in implantation failure^26^ as does knockout of its downstream target COUP-TFII. Heart And Neural Crest Derivatives Expressed 2 (*Hand2,* another P4 regulated transcription factor) is also vital for endometrial receptivity, suppressing the E2-regulated epithelial cell proliferation. Without *Hand2-*mediated suppression of growth factors, epithelial cells continue to proliferate, leading to implantation failure.^27^ An immunophilin *FKBP52* functions as a co-chaperone for the P4 receptor and absence of this causes the endometrium to be non-receptive.^28^ Collectively, these data demonstrated the importance of P4-regualted changes to the endometrium to establish receptivity to implantation.

We have determined that miR-340-5p, miR-542-3p, and miR-671-5p were modified by exposure of endometrial epithelial cells to progesterone *in vitro*. These progesterone-regulated miRNAs changed coordinate with alterations to selected mRNAs, a specific cohort of which they are predicted to target. Following treatment of endometrial epithelial cells with mimics or inhibitors for these three miRNAs we have determined the likely pathways that are P4 regulated by these miRNAs. We have also determined that the overwhelming majority of the proteins altered by these miRNAs are expressed in human endometrial biopsy samples.^21^ Moreover, we have demonstrated that there is a specific miRNA signature in the endometrium of a subset of women that is associated with miscarriage compared to those with a previous pregnancy loss but a subsequent successful pregnancy outcome. Collectively, these data suggest these P4 modified and mammal-specific miRNAs, and the pathways in which their predicted targets function, are important for successful pregnancy and their dysregulation is associated with pregnancy outcome in a subset of women.

Recent studies have highlighted the transcriptional changes that occur in the endometrium during different stages of the menstrual cycle, but data on miRNA regulation of these processes – specifically in the endometrial epithelium - are more limited. miRNA profiling of whole endometrial biopsies (a heterogeneous mix of cell types) from the proliferative and mid-luteal phase of the menstrual cycle has revealed menstrual stage-specific miRNA signatures.^29^ This suggests the regulation of some of these 49 miRNAs is under the control of the steroid hormone environment at sampling. Similarly, our study has shown that P4 regulates expression of 3 of the 13 stem lineage miRNA gene families in endometrial epithelial cells suggesting these may contribute to regulation of the transcriptional response of the endometrium to changes in steroid hormones during the menstrual cycle.

The overall effect of P4 exposure to these epithelial cells altered transcripts involved in the processes of epithelium development and cellular proliferation, as well as functions involved in transcriptional regulation. Previous studies^29^ found that predicted targets of miRNAs upregulated in mid-secretory phase endometrium were found to target cell-cycle regulators, possibly playing a role in cell turnover during the luteal phase. Analysis of the endometrium from women during the receptive stage of the menstrual cycle found ∼21% of differentially expressed genes (653 out of 3,052) directly bind to or are regulated by the progesterone receptor.^30^ The functions related to these transcripts involve regulation of inflammatory response, estrogen response, cell death and interleukin/STAT signaling – some of which we identified as altered in expression following miRNA mimic/inhibitor treatment in our *in vitro* study. For example, addition of mimics for our three miRNAs of interest to Ishikawa cells changes expression of proteins involved in cellular proliferation (Figure 14).

Of the stem lineage miRNAs that were investigated we identified 3 that are modified by P4 *in vitro* in human endometrial epithelial cells, i.e., miR-340-5p, miR-542-3p, and miR-671-5p. We also showed via RNA sequencing that there were coordinate changes to some of the predicted targets of these miRNAs. P4 plays a critical role in endometrial function in a variety of placental mammals. Indeed, exposure of the endometrial epithelial and glandular cells to the sustained action of P4 is required to downregulate its own receptor to facilitate receptivity to implantation – a mechanism that is conserved in all extant placental mammals studied to date.^31^ Analysis of the 473 mRNAs that were predicted targets of all three P4 modified miRNAs and were altered by P4 themselves (Figure 6) were involved in biological processes including cellular proliferation. In our *in vitro* treatment of endometrial epithelial cells with mimics/inhibitors of these P4 modified miRNAs, proteins involved in the biological processes of cell proliferation were also regulated by these miRNAs (Figures 9, 12, and 15 panel C). Collectively, we propose that P4 acting on endometrial epithelial cells modifies expression of miR-340-5p, miR-542-3p, and miR-671-5p. This regulates expression of their targets that are involved in cellular proliferation to modify the endometrial epithelium to establish receptivity to implantation.

In addition to roles in cellular proliferation, these P4-regulated targets were also overrepresented in the molecular functions of ubiquitin-ubiquitin ligase activity and protein serine/threonine kinase activity. These are cellular processes (cell communication, proliferation, differentiation:^32^) that are also regulated by hCG treatment of human endometrial epithelial cells, as well as adhesion of these cells to molecules that make up the extracellular matrix.^33^ We have demonstrated these three miRNAs target proteins that function in adherens, and their assembly (miR-340), inter- and extracellular transport ((miR-340, miR-542, miR-671). This may indicate that P4 primes the endometrium for the further actions of hCG on the epithelium to facilitate secretion and transport of components of uterine luminal fluid and/or implantation – two key functions of the endometrial epithelium.

miRNAs have been shown previously to play a role in both successful pregnancy and pathophysiology. For example, miR-675 expression increases during placental development and inhibition of the expression of miR-675 negatively regulates cell proliferation in a mouse model of overgrowth.^34^ Several miRNAs play a role in trophoblast invasion (mir-16, -29b,−34a, −155, −210:^35^), are serum biomarkers of preeclampsia,^36^ and have been associated with recurrent pregnancy loss (mir-133a:^37^). Of the 13 stem lineage miRNA families investigated in this study, 5 (mir-154, mir-185, mir-188, mir-423, mir-542) have previously been implicated as differentially expressed in the placenta from preeclamptic pregnancies.^38^^,^^39^^,^^40^ Loss of mir-542 has also been implicated in endometrial stromal cell function by enhancing IGFBP1 expression in decidualisation,^41^ but this is the first report to examine expression in an epithelial model. We provide evidence of cell compartment-specific regulation of miRNA expression and their predicted targets – in keeping with the known physiological regulation of the endometrium [1]. To further substantiate this hypothesis, we used mimics and inhibitors to determine *in vitro* endometrial epithelial cell-specific targets of these P4-regulated miRNAs. The *in vitro* targets of these three miRNAs involved processes of transport and cellular localization – key components of endometrial epithelial cell function. P4 stimulates the secretion of components of uterine luminal fluid (ULF) that supports growth and development of the conceptus before formation of the placenta. Although there are limitations to the use of Ishikawa cells they do display some attributes of a receptive endometrial epithelial layer. As this study investigates the stage during which initial attachment takes place, endometrial epithelial cells were used as opposed to endometrial stromal cells as the innermost layer (and site of attachment) of the endometrium is composed of epithelial cells.^42^ More recently there have been developments in organoid models of endometrial glands and assembloids.^43^^,^^44^^,^^45^ Our approach, though a cellular monolayer of epithelium has allowed us to look at the potential mechanism by which these miRNAs may contribute to endometrial function *in vitro* and for the first time allow the comparison of computationally predicted targets with confirmed miRNA targets in this model. We propose that P4-regulated modification of these miRNAs and their targets function to provide components of the ULF that supports early pregnancy. Dysregulation of this process could contribute to RPL. In addition to identifying *in vitro* targets of these mimics, we compared these to computationally predicted targets of these miRNAs and found very limited overlap (Figures 10, 13, and 16). This is not surprising given the high false discovery rate known for target prediction software, of which mirDB is one of the better options.^46^

We identified a difference in one of these P4-regulated stem-lineage miRNAs in endometrial biopsies from women with different pregnancy outcomes. These biopsies were selected at luteal phase as this study aims to investigate endometrial receptivity, luteal phase being the stage at which endometrium is receptive to implantation of an embryo. This is the only physiologically relevant stage for implantation and is when progesterone is at its peak Luteal phase biopsies were selected as we hypothesized that these miRNAs and their targets play a role in receptivity to implantation. Endometrial receptivity is established in the luteal phase and is the physiologically relevant stage to test expression of these candidate miRNAs s.^47^ These samples are a heterogeneous combination of cells from endometrial biopsies. miRNAs have been associated with stromal cell decidualization in the human endometrium^7^ and miRNA profiling of decidua from week 8 (elective termination) compared to those menstrual endometria - found 9 miRNAs to be differentially expressed including one stem lineage miRNA; mir-423.^48^ This link between miRNAs and uterine receptivity was also established in a study of IVF patients who experienced recurrent implantation failure (RIF)^49^ with dysregulation in the expression of a set of 13 miRNAs (not investigated in our study) in endometria of RIF compared to fertile women. Moreover, the protein targets of the 3 P4-modified miRNAs (miR-340, miR-542, and miR-671) were enriched for cell adhesion, cell cycle/proliferation and inter- and-extracellular transport, all functions known to be implicated in embryo development/implantation.^50^ This further supports the important link between miRNAs and endometrial function. miRNA levels in blood plasma from women suffering from recurrent miscarriage to women who were not pregnant also differ including one of the stem linage miRNAs (miR-127-3p).^51^ Although miR-127-3p is not altered by P4 in our study, there are many factors that could contribute to its modification in circulation, and it may be indicative of dysregulation of the endometrium at a different stage of pregnancy.^51^ In our study we have identified a subpopulation of endometrial samples that had aberrant miRNA expression profiles and this may identify a distinct cellular phenotype of endometrial from women who may be at risk of RM in the future. These could potentially provide targets for intervention in future.

In conclusion, we propose that 3 mammal-specific and evolutionarily conserved microRNA genes miR-340-5p, miR-542-3p, and miR-671-5p, with some of their protein targets are regulated by progesterone – the key hormone of pregnancy in the endometrial epithelium in humans. Moreover, the regulation of these miRNAs and their protein targets regulate the function of transport and secretion, and adhesion of the endometrial epithelia required for successful implantation in humans. Dysfunction of these miRNAs (and therefore the targets they regulate) may contribute to endometrial-derived recurrent pregnancy loss in women.

### Limitations of the study

This study uses Ishikawa cells for endometrial epithelial cell treatment with progesterone to be subsequently analyzed by RNA-Seq and Proteomics. Ishikawa cells are a cancer cell line and so may not completely recapitulate the healthy endometrium. Notwithstanding, Ishikawa cells express functional P4 receptors and respond to signaling cues. Comparison of proteomic results to endometrial biopsy RNA-Seq data to confirm that miRNA targets were present in endometrial tissue sought to increase confidence in the results obtained using the cell line.

## STAR★Methods

### Key resources table

**Table:** 

REAGENT or RESOURCE	SOURCE	IDENTIFIER
**Chemicals, peptides, and recombinant proteins**		

Fetal Bovine Serum	Thermo Fisher Scientific (Life Technologies)	10500064
GSP 100X	GIBCO	10378016
DMEM low glucose +pyruvate +phenol red	Thermo Fisher Scientific (Life Technologies)	31885049
Ham’s F12	Thermo Fisher Scientific (Life Technologies)	21765037
PBS	Sigma Aldrich	D8537-500ML
Lipofectamine 2000 Transfection Reagent	Invitrogen	12313563
Progesterone	Sigma Aldrich	P8783-5G
miRCURY Custom PCR Panel	Qiagen	339332
miRIDIAN microRNA Hairpin Inhibitor Negative Control #1, 5 nmol	Horizon Discovery	IN-001005-01-05
miRIDIAN microRNA Mimic Negative Control #1, 5 nmol	Horizon Discovery	CN-001000-01- 05
miRIDIAN microRNA hsa-miR340-5p mimic, 2 nmol	Horizon Discovery	C-301081-01- 0002
miRIDIAN microRNA hsa-miR671-5p mimic, 2 nmol	Horizon Discovery	C-301000-03- 0002
miRIDIAN microRNA hsa-miR542-3p mimic, 2 nmol	Horizon Discovery	C-300866-03- 0002
miRIDIAN microRNA hsa-miR340-5p hairpin inhibitor, 2 nmol	Horizon Discovery	IH-301081-02- 0002
miRIDIAN microRNA hsa-miR542-3p hairpin inhibitor, 2 nmol	Horizon Discovery	IH-300866-05- 0002
miRIDIAN microRNA hsa-miR671-5p hairpin inhibitor, 2 nmol	Horizon Discovery	IH-301000-04- 0002
miRCURY LNA™ RT Kit	Qiagen	339340
miRCURY LNA™ SYBR Green PCR Kit (4000)	Qiagen	339347
Qiazol® **Lysis Reagent**	Qiagen	79306
miRNeasy Mini Kit (50)	Qiagen	217004
RNase-Free DNase Set (50)	Qiagen	79254
High Capacity c DNA Rt Kit	Applied Biosystems	4368814

**Deposited data**		

Proteomic data	ProteomeXchange	PXD036109
RNASeq data	GEO	GSE211151

**Experimental models: Cell lines**		

Ishikawa Cells	Cell culture collections	ECACC # 99040201

**Oligonucleotides**		

Designed primers	IDT	

**Software and algorithms**		

Graphpad Prism		
Webgestalt	www.webgestalt.org	

### Resource availability

#### Lead contact

Lead contact and further information requests should be directed to and will be fulfilled by the lead contact, Niamh Forde (n.forde@leeds.ac.uk).

#### Materials availability

This study did not generate new unique reagents.

### Experimental model and subject details

#### Human subjects

All human biopsies were from female patients. The ages of individuals were in the Infertile + live birth group ranged between 25-39 (specifically: 31, 38, 25, 38, 32, 31, 34, 37, 36, 27, 39, 27), for the miscarriage + live birth group ranging between 28-39 (specifically: 34, 28, 30, 33, 36, 31, 27, 34, 38, 32, 39, 36), and in the miscarriage + miscarriage ranging between (specifically: 35, 33, 25, 31, 36, 35, 31, 31, 30, 34, 28, 36). The study was approved by the NHS Research Ethics Committee, Hammersmith and Queen Charlotte’s & Chelsea Research Ethics Committee (1997/5065), and Tommy’s National Reproductive Health Biobank (REC reference: 18/WA/0356). Human endometrial samples were obtained with written informed consent and in accordance with The Declaration of Helsinki (2000) guidelines. Biopsies were obtained from women attending the Implantation Clinic, a dedicated research clinic at University Hospitals Coventry and Warwickshire (UHCW) National Health Service Trust.

#### Cell lines

Ishikawa cells (adenocarcinoma endometrial epithelial cells) (ECACC # 99040201).

### Method details

Unless otherwise stated all consumables were sourced from Sigma Aldrich (UK).

#### Cell culture

Human immortalised endometrial epithelial cells (Ishikawa cells - ECACC: #99040201) were cultured (50:50 DMEM/F12, 0.5% GSP, 10% charcoal-stripped FBS) at 37 °C and in 5% CO2. Cells were cultured to 70% confluence and seeded into 6-well plates. The cultures were treated with either 1) basal media (as above), 2) vehicle control (70% ethanol, EtOH) or 3), 0.1 mg/ml P4, 4) 1.0 mg/ml P4, or 5) 1.0 mg/ml P4, for 24 hours. Following treatment, cells were harvested by trypsinization and lysed in QIAzol Lysis Reagent. RNA was extracted using the miRNeasy Mini RNA Extraction kit (Qiagen, UK) as per manufacturer’s instructions.

#### miRNA expression analysis and target prediction

Reverse transcription was carried out using the miRCURY LNA™1 RT Kit (Qiagen) using 10 ng of RNA according to manufacturer’s instructions, alongside reverse transcriptase negative controls. Expression analysis was performed using the miRCURY LNA™ SYBR Green PCR Kit 4000 (Qiagen) and a custom designed plate including miRNAs of interest and controls for normalisation (5S rRNA, U6 snRNA, UniSP3, and UniSP6). Thermal cycling and detection were performed on a ROCHE Light Cycler® 480 II using the following parameters: 2minat 90 °C, followed by 45 cycles of 10secsat 95°C & 60secsat 56°C, and melting curve analysis for 60minat 95 °C. Cq values were determined and normalised to the geometric mean of normalisers. Data were analysed using ANOVA in Graphpad Prism to determine differences (determined as significant when p <0.05).

Predicted targets of miRNAs with P4-dependent expression were identified using TargetScan 7.2,^52^ with output filtered based on the level of complementary binding between the miRNA seed region and target site: targets with 7mer or 8mer complementary binding to the seed region were selected, since the high number of consecutive matches is indicative of an efficient binding site.^53^ Ensembl transcript IDs provided by the TargetScan output were then mapped to gene names using Ensembl BioMart (release 98).^54^

#### q-RT-PCR of predicted miRNA targets

Reverse transcription from 10 μg of RNA was carried out using the High-Capacity cDNA Reverse Transcription Kit (Applied Biosystems) as per the manufacturer’s protocol. Primers were designed for selected transcripts using Primer-BLAST (Table 1) and 20 μM stock dilutions were prepared. PCR reactions were prepared in 20 μl volumes, containing 5 μl Roche SYBR Green Master Mix, 0.5 μl of each forward and reverse primer and 2 μl cDNA. Each sample was analysed in duplicate using the Lightcycler 480 II (Roche) as follows: 95 °C for 5 mins, 45 cycles of 95 °C for 10 secs, then 60 °C for 10 secs and finally 72 °C for secs, followed by melt curve analysis. Data were analysed using the comparative CT method (2-ΔΔCt)^55^ normalised to the geometric mean of ACTB, GAPDH and PPIA. Significant differences were determined using ANOVA analysis in Graphpad Prism software, including Dunnett’s multiple comparisons test, to identify effects of treatment between each treatment concentration and the vehicle control. Statistical significance was assumed when p < 0.05 after multiple testing correction.

#### RNA sequencing analysis

Total RNA (100 ng) was used for library preparation using the Illumina TruSeq® Stranded Total kit, according to the manufacturer’s guidelines. The library quality, size range, and sequencing adaptor dimer contamination was assessed with an Agilent TapeStation 2200 using the DNA broad range kit. Excess sequencing adaptor dimer was removed by AxyPrep Mag PCR clean up Kit if present. Final libraries were quantified by the Qubit dsDNA assay kit and Qubit fluorometer (Life Technologies) before creating an equimolar pool of the libraries. The libraries were sequenced by the Next Generation Sequencing Facility at the University of Leeds, using the NextSeq 500 (Illumina, California, USA) to generate single end 75 bp reads. Reads were trimmed by Cutadapt^56^ and filtered using fastq_quality_filter as part of FASTX-toolkit (http://hannonlab.cshl.edu/fastx_toolkit/) with parameters including “-q 20” and “-p 90”. The reference genome and gene annotation files of *Homo sapiens* were retrieved from GENCODE (release 31) (https://www.gencodegenes.org/).^57^ Read mapping was performed by means of Subread aligners in Rsubread package^58^ with only uniquely mapped reads reported in the final alignment and read summarisation. Principal Component Analysis (PCA) plot was carried out using all protein-coding genes and long noncoding RNAs with RPKM value ≥1 in at least one sample. Subsequently, log2(RPKM+1) transformation and quantile normalization were applied.

Quantification was carried out by featureCounts function in R. Significant differences in gene expression were identified using DESeq2^59^ with the following cut-offs: log2FoldChange >1 (or <−1) and an FDR-adjusted p value of <0.05. After this analysis, only protein-coding genes were retained based on the gene biotype labels. Overrepresentation enrichment analysis of DEGs were identified using WebGestalt (http://www.webgestalt.org/)^60^ for gene ontology (GO) terms and pathways, with a significance enrichment level set at an FDR <0.05.

#### miRNA mimic/inhibitor experiments

Ishikawa cells were plated (∼200,000 cells/well) 24 hours prior to treatment. At the time of treatment, cells were washed 3 times in PBS and 1.6 ml antibiotic & serum-free media added per well. The following treatments (n = 3 biological replicates) were added 1) control medium, 2) Transfection reagent (Lipofectamine 2000), 3) miRNA mimic of interest (80 pmol/well), 4) miRNA inhibitor of interest, 5) miRNA non-targeting mimic, 6) miRNA non-targeting inhibitor. Cells were incubated for 48 hours before media was removed, washed with PBS, and protein extracted using a RIPA buffer with protease inhibitors by passing the cells through a 21′G needle and syringe.

#### Proteomic analysis

Fifty μg of protein from each sample, described above, was digested with trypsin (1.25 μg trypsin; 37°C, overnight), labelled with Tandem Mass Tag (TMT) eleven plex reagents according to the manufacturer’s protocol (Thermo Fisher Scientific, Loughborough, LE11 5RG, UK) and the labelled samples pooled. One hundred μg of the sample pool was desalted using a SepPak cartridge according to the manufacturer’s instructions (Waters, Milford, Massachusetts, USA). Eluate from the SepPak cartridge was evaporated to dryness and resuspended in buffer A (20 mM ammonium hydroxide, pH 10) prior to fractionation by high pH reversed-phase chromatography using an Ultimate 3000 liquid chromatography system (Thermo Fisher Scientific). In brief, the sample was loaded onto an XBridge BEH C18 Column (130Å, 3.5 μm, 2.1 mm × 150 mm, Waters, UK) in buffer A and peptides eluted with an increasing gradient of buffer B (20 mM Ammonium Hydroxide in acetonitrile, pH 10) from 0–95% over 60 mins. The resulting fractions (15 in total) were evaporated to dryness and resuspended in 1% formic acid prior to analysis by nano-LC MSMS using an Orbitrap Fusion Lumos mass spectrometer (Thermo Scientific). In brief, peptides in 1% (vol/vol) formic acid were injected onto an Acclaim PepMap C18 nano-trap column (Thermo Scientific). After washing with 0.5% (vol/vol) acetonitrile, and 0.1% (vol/vol) formic acid, peptides were resolved on a 250 mm × 75 μm Acclaim PepMap C18 reverse phase analytical column (Thermo Scientific) over a 150 mins organic gradient, using 7 gradient segments (1–6% solvent B over 1 mins, 6–15% B over 58 mins, 15–32% B over 58 mins, 32–40% B over 5 mins, 40–90% B over 1 mins, held at 90% B for 6 mins and then reduced to 1% B over 1 mins) with a flow rate of 300 nl min−1. Solvent A was 0.1% formic acid and Solvent B was aqueous 80% acetonitrile in 0.1% formic acid. Peptides were ionized by nano-electrospray ionization at 2.0 kV using a stainless-steel emitter with an internal diameter of 30 μm (Thermo Scientific) and a capillary temperature of 300 °C. All spectra were acquired using an Orbitrap Fusion Lumos mass spectrometer controlled by Xcalibur 3.0 software (Thermo Scientific) and operated in data-dependent acquisition mode using an SPS-MS3 workflow. FTMS1 spectra were collected at a resolution of 120000, with an automatic gain control (AGC) target of 200000 and a max injection time of 50 ms. Precursors were filtered with an intensity threshold of 5000, according to charge state (to include charge states 2–7) and with monoisotopic peak determination set to Peptide. Previously interrogated precursors were excluded using a dynamic window (60 secs +/−10 ppm). The MS2 precursors were isolated with a quadrupole isolation window of 0.7. m/z. ITMS2 spectra were collected with an AGC target of 10,000, max injection time of 70 ms and CID collision energy of 35%. For FTMS3 analysis, the Orbitrap was operated at 50,000 resolution with an AGC target of 50,000 and a max injection time of 105 ms. Precursors were fragmented by high energy collision dissociation (HCD) at a normalised collision energy of 60% to ensure maximal TMT reporter ion yield. Synchronous Precursor Selection (SPS) was enabled to include up to 10 MS2 fragment ions in the FTMS3 scan.

The raw data files were processed and quantified using Proteome Discoverer software v2.1 (Thermo Scientific) and searched against the UniProt Human database (downloaded January 2021: 169,297 entries) using the SEQUEST HT algorithm. Peptide precursor mass tolerance was set at 10 ppm, and MS/MS tolerance was set at 0.6 Da. Search criteria included oxidation of methionine (+15.995 Da), acetylation of the protein N-terminus (+42.011 Da) and Methionine loss plus acetylation of the protein N-terminus (−89.03 Da) as variable modifications and carbamidomethylation of cysteine (+57.021 Da) and the addition of the TMT mass tag (+229.163 Da) to peptide N-termini and lysine as fixed modifications. Searches were performed with full tryptic digestion and a maximum of 2 missed cleavages were allowed. The reverse database search option was enabled and all data was filtered to satisfy false discovery rate (FDR) of 5%. Protein groupings were determined by PD2.1, with minor modifications to allow inference of biological trends without any loss in the quality of identification or quantification. The MS data were searched against the human Uniprot database retrieved on 2021-01-14 and updated with additional annotation information on 2021-11-15. The protein abundances were normalised within each sample to total peptide amount, then Log2 transformed to bring them closer to a normaldistribution. Statistical significance was then determined using paired t-tests between the conditions of interest with FDR corrected pvalues obtained using the Benjamini-Hochberg method.

#### Analysis of miRNAs in clinical samples

Human endometrial samples were obtained with written informed consent and in accordance with The Declaration of Helsinki (2000) guidelines. Biopsies were obtained from women attending the Implantation Clinic, a dedicated research clinic at University Hospitals Coventry and Warwickshire (UHCW) National Health Service Trust. Samples were timed relative to the pre-ovulatory LH surge, and obtained using a Wallach Endocell™ endometrial sampler. Following luteal phase endometrial biopsy, pieces of tissue (∼2 mm) were preserved in RNA-later and stored at −80°C prior to analyses. Tissues were homogenised in 1 ml STAT-60 and RNA isolated according to the manufacturer’s instructions (AMS-Bio, UK). RNA was resuspended in 50μl TE buffer (pH 8.0) and assessed by Nanodrop ND-1000 spectrophotometer, before storage at −80°C. Samples underwent reverse transcription and miRNA expression analysis as described above. Expression of miRNAs of interest was measured in patients suffering from infertility and a subsequent live birth (IF+LB; n = 12), patients with miscarriage and a subsequent live birth (M+LB; n = 12), and patients suffering miscarriage and a subsequent miscarriage (M+M; n = 12).

### Quantification and statistical analysis

Statistical significance was defined by p <0.05 in all experiments. Statistical analysis of miRNA expression levels (Figures 1 and 18) was determined by an ANOVA. Expression of miRNA in response to P4 (Figure 1) has n =3 biological repeats of Ishikawa cells. Differences between groups are denoted by difference in letters (a, b, c, d). Expression of miRNAs in clinical human endometrial biopsies consists of n = 12 patients per group. Graphpad Prism was used for graphical representation of data. In Figure 2 differences in expression compared were determined using ANOVA with differences depicted by different subscripts (a, b, c, d) when p < 0.05.

RNA Seq data was analysed by quantification carried out by featureCounts function in R. Significant differences in gene expression were identified using DESeq2 (28) with the following cut-offs: log2FoldChange >1 (or <−1) and an FDR-adjusted p value of <0.05. Overrepresentation analyses were performed using WebGestAlt with FDR <0.05.

Proteomic statistical analysis (Figures 9, 10, 11, 12, 13, 14, 15, 16, and 17) was achieved using paired t-tests and a Benjamini Hochberg correction. Proteomic samples were carried out in n = 3 biological replicates of Ishikawa cells. Graphpad Prism was used for graphical representation of data.

Statistical significance of q-RT-PCR of predicted miRNA targets was carried out using the comparative CT method (2-ΔΔCt) (24) normalised to the geometric mean of ACTB, GAPDH and PPIA. Significance differences were determined using ANOVA analysis in Graphpad Prism software, including Dunnett’s multiple comparisons test.

## Data Availability

• All RNA sequencing and proteomic data are uploaded in publicly available data repositories - RNA sequencing data is available on GEO under accession number GEO:GSE211151. • The mass spectrometry proteomics data have been deposited to the ProteomeXchange Consortium via the PRIDE partner repository with the dataset identifier ProteomeXchange:PXD036109. • This paper does not report original code and any additional information required to reanalyse the data reported in this paper is available from the lead contact upon request.

## Supplemental information

Document S1. Figures S1–S9

Table S1. Genes differentially expressed in endometrial epithelial cells following treatment with P4, related to Figure 3 Gene ID, gene name, gene type, gene id, baseMean expression value, log2FoldChange, lfcSE, stat, pvalue, and padj for transcripts identified as differentially expressed in Ishikawa cells (n = 3 biological replicates) determined by RNA sequencing. Values reflect changes in P4 treated cells (10 μg/ml) compared to vehicle controls. Raw data are accessible at GEO under accession number GSE211151.

Table S2. Overrepresented Gene Ontologies (GO) for DEGs in endometrial epithelial cells following treatment with P4 *in vitro*, related to Figure 4 Overrepresented gene ontologies (GO) terms for biological processes (BP), description, GO term size, expected and degree of enrichment for DEGs identified following RNA sequencing of P4 treated Ishikawa cells (n = 3 biological replicates) compared to vehicle controls. Significance of enrichment, p-value, FDR-adjusted p-value and gene IDs are provided. GO overrepresentation analyses were carried out using webgestalt.

Table S3. Overrepresented Gene Ontologies (GO) for DEGs in endometrial epithelial cells following treatment with P4 *in vitro*, related to Figure 4 Overrepresented gene ontologies (GO) terms for cell component (CC), description, GO term size, expected and degree of enrichment for DEGs identified following RNA sequencing of P4 treated Ishikawa cells (n = 3 biological replicates) compared to vehicle controls. Significance of enrichment, p-value, FDR-adjusted p-value and gene IDs are provided. GO overrepresentation analyses were carried out using webgestalt.

Table S4. Overrepresented Gene Ontologies (GO) for DEGs in endometrial epithelial cells following treatment with P4 *in vitro*, related to Figure 4 Overrepresented gene ontologies (GO) terms for molecular function (MF), description, GO term size, expected and degree of enrichment for DEGs identified following RNA sequencing of P4 treated Ishikawa cells (n = 3 biological replicates) compared to vehicle controls. Significance of enrichment, p-value, FDR-adjusted p-value and gene IDs are provided. GO overrepresentation analyses were carried out using webgestalt.

Table S5. Enriched REACTOME pathways for DEGs in endometrial epithelial cells following treatment with P4 *in vitro*, related to Figure 5 Enriched REACTOME pathways description, GO term size, expected and degree of enrichment for DEGs identified following RNA sequencing of P4 treated Ishikawa cells (n = 3 biological replicates) compared to vehicle controls. Significance of enrichment, p-value, FDR-adjusted p-value and gene IDs are provided. GO overrepresentation analyses were carried out using webgestalt.

Table S6. Enriched KEGG pathways for DEGs in endometrial epithelial cells following treatment with P4 *in vitro*, related to Figure 5 Enriched KEGG pathways description, GO term size, expected and degree of enrichment for DEGs identified following RNA sequencing of P4 treated Ishikawa cells (n = 3 biological replicates) compared to vehicle controls. Significance of enrichment, p-value, FDR-adjusted p-value and gene IDs are provided. GO overrepresentation analyses were carried out using webgestalt.

Table S7. Venn diagram analysis of P4-regualted mRNA and predicted miRNA targets, related to Figure 6 Venn diagram analysis of list of transcripts that are common or unique to P4-modified mRNAs determined by RNA sequencing that were altered in Ishikawa cells treated with P4 for 24 h (n = 3 biological replicates) or are predicted targets of three stem lineage. miRNAs that are also altered by P4 treatment of endometrial epithelial cells.

Table S8. Overrepresented Gene Ontologies (GO) for DEGs in endometrial epithelial cells following treatment with P4 *in vitro* that are also predicted targets of P4-modified miRNAs, related to Figure 7 Overrepresented gene ontologies (GO) terms for biological processes (BP), description, GO term size, expected and degree of enrichment for DEGs identified following RNA sequencing of P4 treated Ishikawa cells (n = 3 biological replicates) compared to vehicle controls that are also predicted targets (as determined by TargetScan) of three miRNA that are P4-modified. Significance of enrichment, p-value, FDR-adjusted p-value and gene IDs are provided. GO overrepresentation analyses were carried out using webgestalt.

Table S9. Overrepresented Gene Ontologies (GO) for DEGs in endometrial epithelial cells following treatment with P4 *in vitro* that are also predicted targets of P4-modified miRNAs, related to Figure 7 Overrepresented gene ontologies (GO) terms for cellular components (CC), description, GO term size, expected and degree of enrichment for DEGs identified following RNA sequencing of P4 treated Ishikawa cells (n = 3 biological replicates) compared to vehicle controls that are also predicted targets (as determined by TargetScan) of three miRNA that are P4-modified. Significance of enrichment, p-value, FDR-adjusted p-value and gene IDs are provided. GO overrepresentation analyses were carried out using webgestalt.

Table S10. Overrepresented Gene Ontologies (GO) for DEGs in endometrial epithelial cells following treatment with P4 *in vitro* that alre also predicted targets of P4-modified miRNAs, related to Figure 7 Overrepresented gene ontologies (GO) terms for molecular function (MF), description, GO term size, expected and degree of enrichment for DEGs identified following RNA sequencing of P4 treated Ishikawa cells (n = 3 biological replicates) compared to vehicle controls that are also predicted targets (as determined by Targetscan) of three miRNA that are P4-modified. Significance of enrichment, p-value, FDR-adjusted p-value and gene IDs are provided. GO overrepresentation analyses were carried out using webgestalt.

Table S11. Enriched REACTOME pathways for DEGs in endometrial epithelial cells following treatment with P4 *in vitro* that are also predicted targets of P4-modified miRNAs, related to Figure 8 Enriched REACTOME pathways, description, GO term size, expected and degree of enrichment for DEGs identified following RNA sequencing of P4 treated Ishikawa cells (n = 3 biological replicates) compared to vehicle controls that are also predicted targets (as determined by Targetscan) of three miRNA that are P4-modified. Significance of enrichment, p-value, FDR-adjusted p-value and gene IDs are provided. GO overrepresentation analyses were carried out using webgestalt.

Table S12. Enriched KEGG pathways for DEGs in endometrial epithelial cells following treatment with P4 *in vitro* that are also predicted targets of P4-modified miRNAs, related to Figure 8 Enriched KEGG pathways, description, GO term size, expected and degree of enrichment for DEGs identified following RNA sequencing of P4 treated Ishikawa cells (n = 3 biological replicates) compared to vehicle controls that are also predicted targets (as determined by TargetScan) of three miRNA that are P4-modified. Significance of enrichment, p-value, FDR-adjusted p-value and gene IDs are provided. GO overrepresentation analyses were carried out using webgestalt.

Table S13. List of proteins significantly altered in abundance in response to miR-340-5p mimic, inhibitor, both (p<0.05), n = 3, related to Figure 9

Table S14. List of proteins significantly altered in abundance in response to miR-340-5p mimic and/or inhibitor, miRDB Predicted targets and P4 regulated genes which are predicted targets of miR-340-5p by TargetScan (p<0.05), n = 3, related to Figure 10

Table S15. S15A: List of gene names in segments of Venn diagram related to Figure 11. S15B) RNA-Seq data for human endometrial biopsies for mRNAs matching 1,192 proteins changed in response to miR-340-5p mimic, related to Figure 11. S15C) RNA-Seq data for human endometrial biopsies for mRNAs matching 267 proteins changed in response to miR-340-5p inhibitor, related to Figure 11. S15D) RNA-Seq data for human endometrial biopsies for mRNAs matching 59 proteins changed in response to miR-340-5p mimic AND inhibitor, related to Figure 11. 15A: List of gene names in segments of Venn diagram related to Figure 11.15B) RNA-Seq data for human endometrial biopsies for mRNAs matching 1,192 proteins changed in response to miR-340-5p mimic, related to Figure 11.15C) RNA-Seq data for human endometrial biopsies for mRNAs matching 267 proteins changed in response to miR-340-5p inhibitor, related to Figure 11.15D) RNA-Seq data for human endometrial biopsies for mRNAs matching 59 proteins changed in response to miR-340-5p mimic AND inhibitor, related to Figure 11.

Table S16. List of proteins significantly altered in abundance in response to miR-542-3p mimic, inhibitor, both (p<0.05), n = 3, related to Figure 12

Table S17. List of proteins significantly altered in abundance in response to miR-542-3p mimic and/or inhibitor, miRDB Predicted targets and P4 regulated genes which are predicted targets of miR-542-3p by TargetScan (p<0.05), n = 3, related to Figure 13

Table S18. S18A) List of gene names in segments of Venn diagram related to Figure 14. S18B) RNA-Seq data for human endometrial biopsies for mRNAs matching 1,080 proteins changed in response to miR-542-3p mimic, related to Figure 14. S18C) RNA-Seq data for human endometrial biopsies for mRNAs matching 714 proteins changed in response to miR-542-3p inhibitor, related to Figure 14. S18D) RNA-Seq data for human endometrial biopsies for mRNAs matching 181 proteins changed in response to miR-542-3p mimic AND inhibitor, related to Figure 14. 18A) List of gene names in segments of Venn diagram related to Figure 14.18B) RNA-Seq data for human endometrial biopsies for mRNAs matching 1,080 proteins changed in response to miR-542-3p mimic, related to Figure 14.18C) RNA-Seq data for human endometrial biopsies for mRNAs matching 714 proteins changed in response to miR-542-3p inhibitor, related to Figure 14.18D) RNA-Seq data for human endometrial biopsies for mRNAs matching 181 proteins changed in response to miR-542-3p mimic AND inhibitor, related to Figure 14.

Table S19. List of proteins significantly altered in abundance in response to miR-671-5p mimic, inhibitor, both (p<0.05), n = 3, related to Figure 15

Table S20. List of proteins significantly altered in abundance in response to miR-671-5p mimic and/or inhibitor, miRDB Predicted targets and P4 regulated genes which are predicted targets of miR-671-5p by TargetScan (p<0.05), n = 3, related to Figure 16

Table S21. S21A) List of gene names in segments of Venn diagram related to Figure 17. S21B) RNA-Seq data for human endometrial biopsies for mRNAs matching 1,063 proteins changed in response to miR-671-5p mimic, related to Figure 17. S21C) RNA-Seq data for human endometrial biopsies for mRNAs matching 362 proteins changed in response to miR-671-5p inhibitor, related to Figure 17. S21D) RNA-Seq data for human endometrial biopsies for mRNAs matching 87 proteins changed in response to miR-671-5p mimic AND inhibitor, related to Figure 17. Table 21A: List of gene names in segments of Venn diagram related to Figure 17. Table S21B: RNA-Seq data for human endometrial biopsies for mRNAs matching 1,063 proteins changed in response to miR-671-5p mimic, related to Figure 17. Table S21C: RNA-Seq data for human endometrial biopsies for mRNAs matching 362 proteins changed in response to miR-671-5p inhibitor, related to Figure 17. Table S21D: RNA-Seq data for human endometrial biopsies for mRNAs matching 87 proteins changed in response to miR-671-5p mimic AND inhibitor, related to Figure 17.

Table S22. List of proteins significantly altered in abundance in response to non-targeting mimic and miR-340-5p mimic. Final column lists proteins solely altered by miR-340-5p mimic once off target effects are removed (p<0.05), n = 3, related to Figure 9

Table S23. List of proteins significantly altered in abundance in response to non-targeting inhibitor and miR-340-5p inhibitor, related to Figure 9 Final column lists proteins solely altered by miR-340-5p inhibitor once off target effects are removed (p<0.05), n = 3.

Table S24. List of proteins significantly altered in abundance in response to miR-340-5p mimic and/or inhibitor, miRDB predicted targets of miR-340-5p, related to Figure 10 Final column lists genes common to altered proteins in miR-340-5p mimic and/or inhibitor and a miRDB predicted target (p<0.05), n = 3.

Table S25. List of proteins significantly altered in abundance in response to non-targeting mimic and miR-542-3p mimic, related to Figure 12 Final column lists proteins solely altered by miR-542-3p mimic once off target effects are removed (p<0.05), n = 3.

Table S26. List of proteins significantly altered in abundance in response to non-targeting inhibitor and miR-542-3p inhibitor, related to Figure 12 Final column lists proteins solely altered by miR-542-3p inhibitor once off target effects are removed (p<0.05), n = 3.

Table S27. List of proteins significantly altered in abundance in response to miR-542-3p mimic and/or inhibitor, miRDB predicted targets of miR-542-3p, related to Figure 13 Final column lists genes common to altered proteins in miR-542-3p mimic and/or inhibitor and a miRDB predicted target (p<0.05), n = 3.

Table S29. List of proteins significantly altered in abundance in response to non-targeting mimic and miR-671-5p mimic, related to Figure 15 Final column lists proteins solely altered by miR-671-5p inhibitor once off target effects are removed (p<0.05), n = 3.

Table S28. List of proteins significantly altered in abundance in response to non-targeting inhibitor and miR-671-5p inhibitor, related to Figure 15 Final column lists proteins solely altered by miR-671-5p mimic once off target effects are removed (p<0.05), n = 3

Table S30. List of proteins significantly altered in abundance in response to miR-671-5p mimic and/or inhibitor, miRDB predicted targets of miR-671-5p, related to Figure 16 Final column lists genes common to altered proteins in miR-671-5p mimic and/or inhibitor and a miRDB predicted target (p<0.05), n = 3.
