# Supplementary material for: MicroRNAs emerging coordinate with placental mammals alter pathways in endometrial epithelia important for endometrial function
Source: iScience. 2023 Mar 4;26(4):106339. doi: 10.1016/j.isci.2023.106339 (PMC10034127; doi:10.1016/j.isci.2023.106339)
Supplement: Document S1. Figures S1–S9 [file mmc1.pdf]

iScience, Volume *26*

Supplemental information

MicroRNAs emerging coordinate with placental mammals alter pathways in endometrial epithelia important for endometrial function

Laura Hume, Jessica C. Edge, Haidee Tinning, Dapeng Wang, Alysha S. Taylor, Vladimir Ovchinnikov, Annika V. Geijer-Simpson, Pavle Vrljicak, Jan J. Brosens, Emma S. Lucas, Nigel A.B. Simpson, Jayne Shillito, Karen Forbes, Mary J. O'Connell, and Niamh Forde

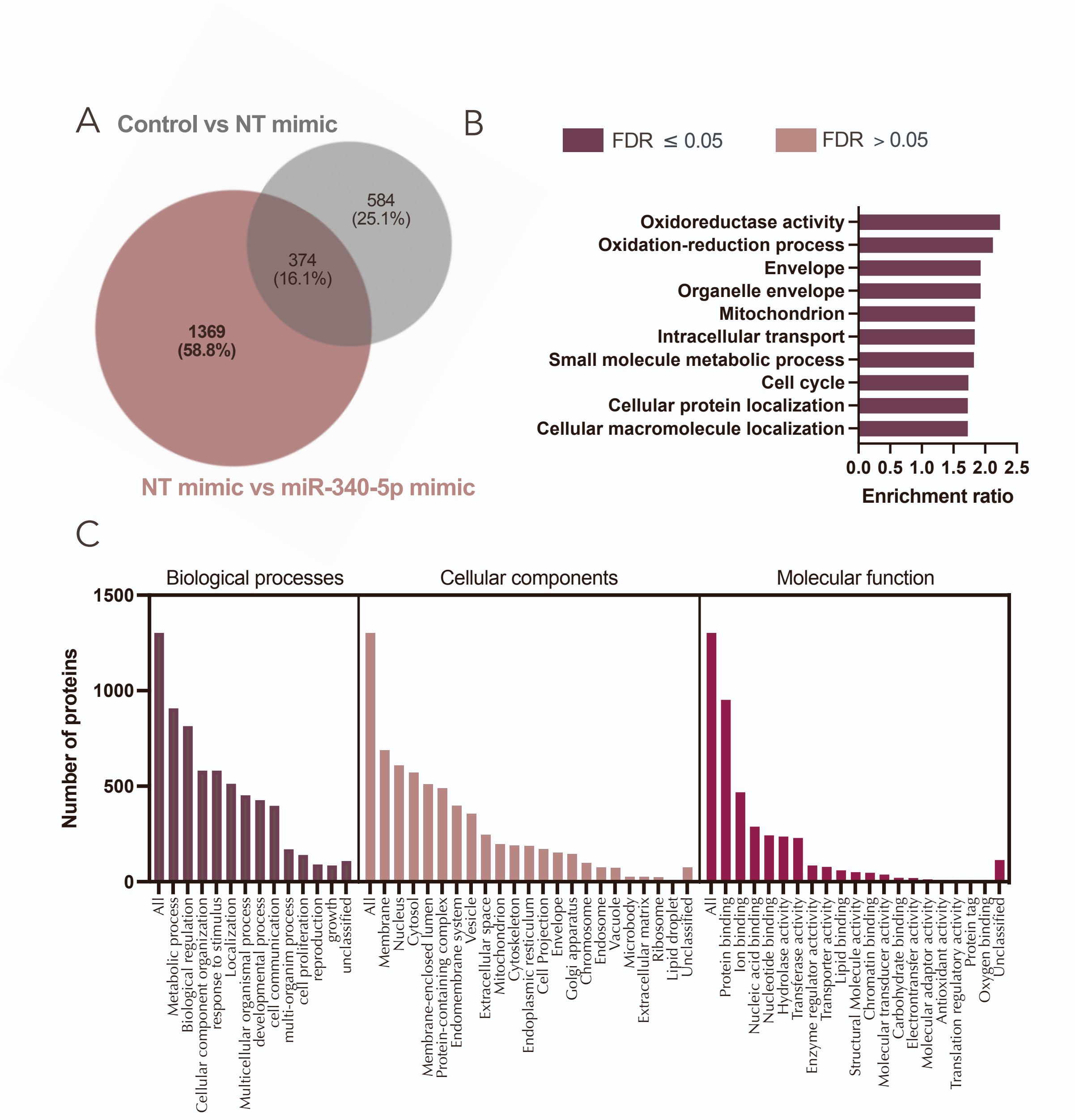

Supplementary Figure 1: *Proteins altered in Ishikawa cells following treatment with miR-340-5p mimic related to Figure 9.* **A)** Venn diagram depicting total number of significantly differentially expressed proteins (p<0.05) following transfection of Ishikawa cells (n=3 biological replicates) with miR-340-5p mimic (LHS) and non-targeting (NT) mimic (RHS). **B)** Enriched KEGG pathways associated with miR-340-5p mimic regulated proteins. **C)** WebGestalt overrepresentation analysis of biological process, cellular component and molecular function categories for identified significantly differentially expressed proteins (p<0.05) in response to miR-340-5p mimic.

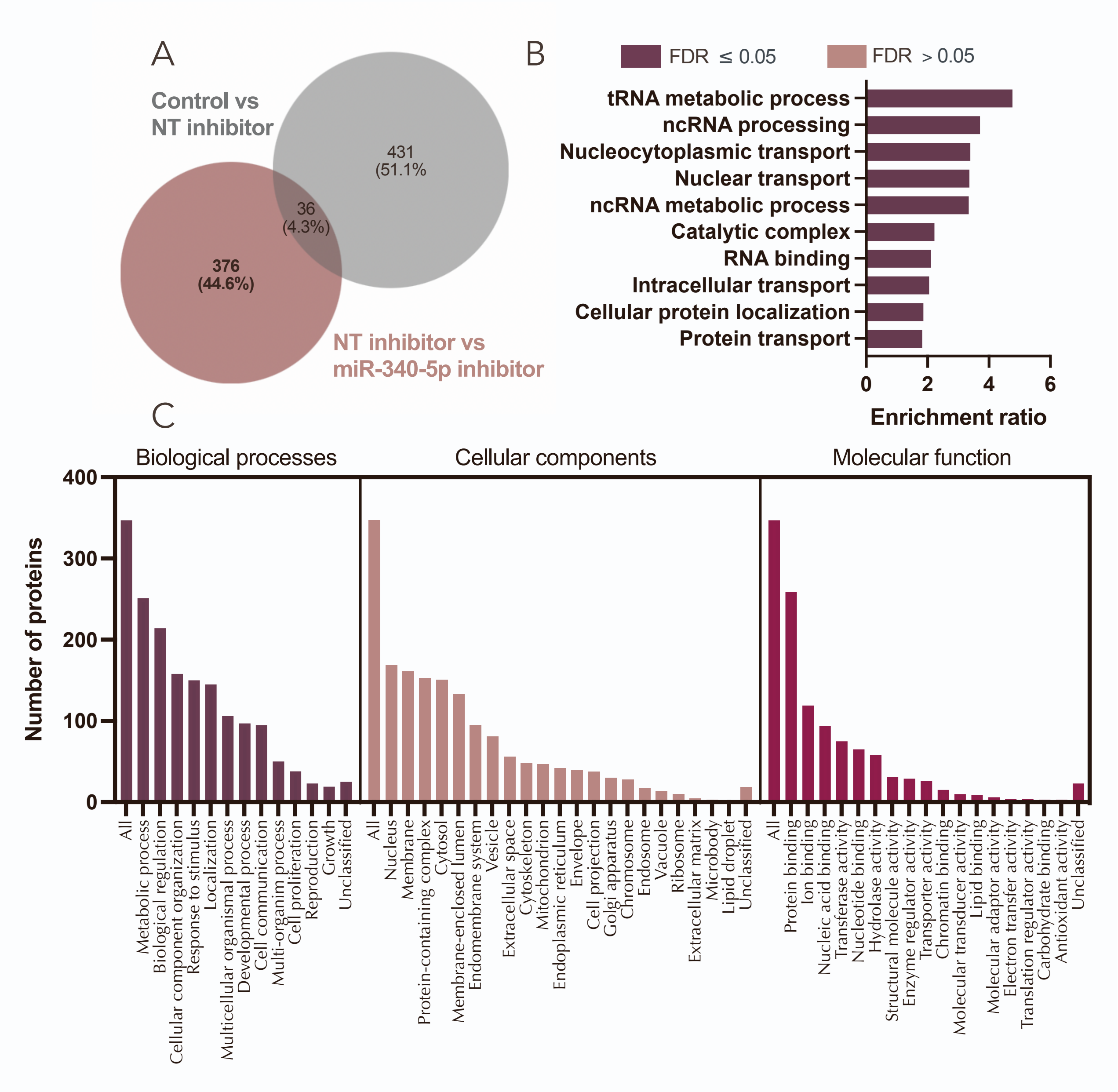

Supplementary Figure 2: *Proteins altered in Ishikawa cells following treatment with miR-340-5p inhibitor related to Figure 9.* **A)** Venn diagram depicting total number of significantly differentially expressed proteins (p<0.05) following transfection of Ishikawa cells (n=3 biological replicates) with miR-340-5p inhibitor (LHS) and non-targeting (NT) inhibitor (RHS). **B)** Enriched KEGG pathways associated with miR-340-5p inhibition regulated proteins. **C)** WebGestalt overrepresentation analysis of biological process, cellular component and molecular function categories for identified significantly differentially expressed proteins (p<0.05) in response to miR-340-5p inhibitor.

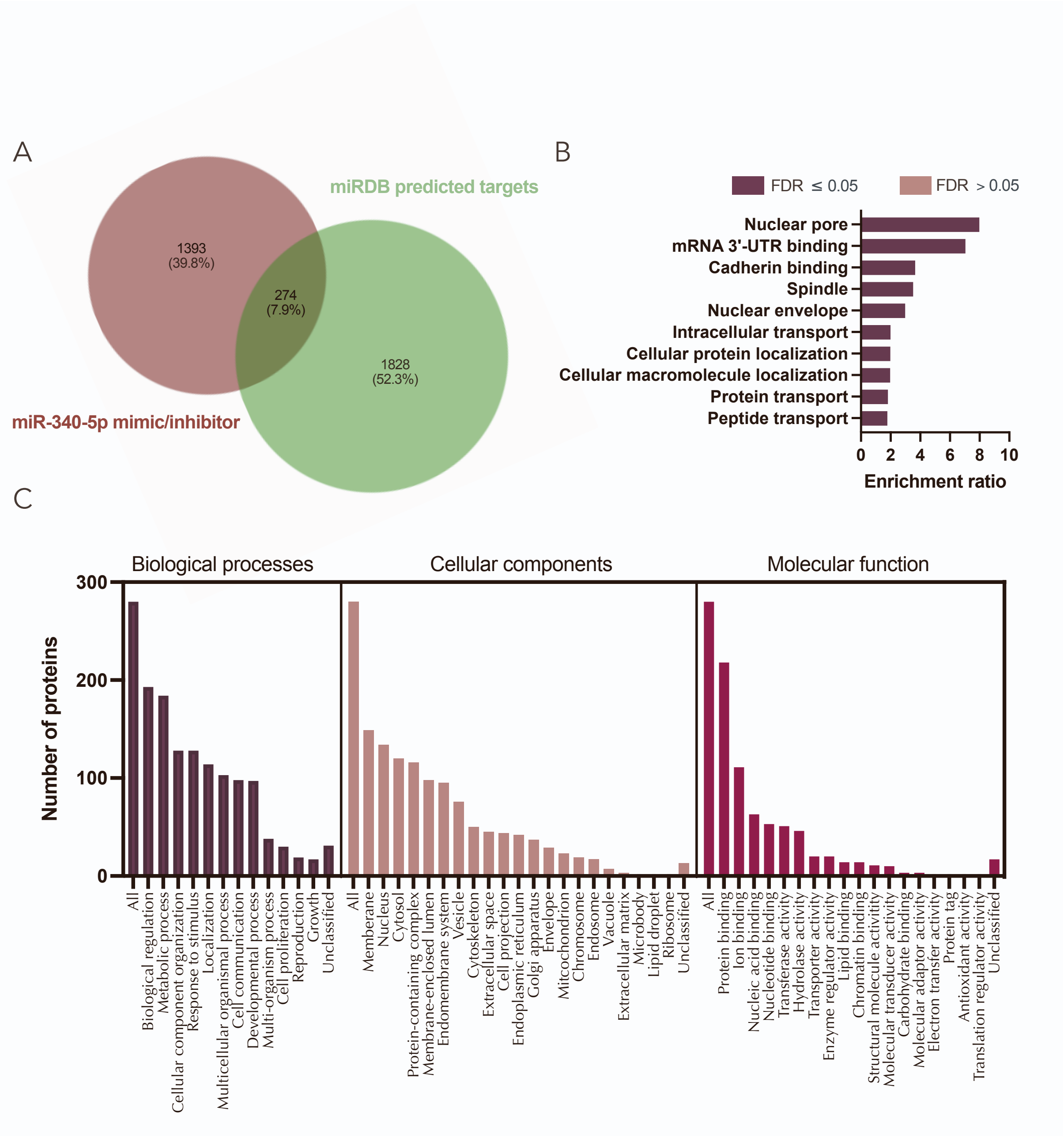

Supplementary Figure 3: *Proteins altered in Ishikawa cells following treatment with miR-340-5p mimic and inhibitor compared to mirDB predicted targets related to Figure 9.* **A)** Venn diagram depicting total number of significantly differentially expressed proteins (p<0.05) following transfection of Ishikawa cells (n=3 biological replicates) with miR-340-5p mimic or inhibitor vs non-targeting controls and mirDB predicted targets. **B)** Enriched KEGG pathways associated with miR-340-5p mimic and inhibition regulated proteins and predicted target overlap. **C)** WebGestalt overrepresentation analysis of biological process, cellular component

and molecular function categories for identified significantly differentially expressed proteins (p<0.05) in response to miR-340-5p mimic and inhibition and predicted targets.

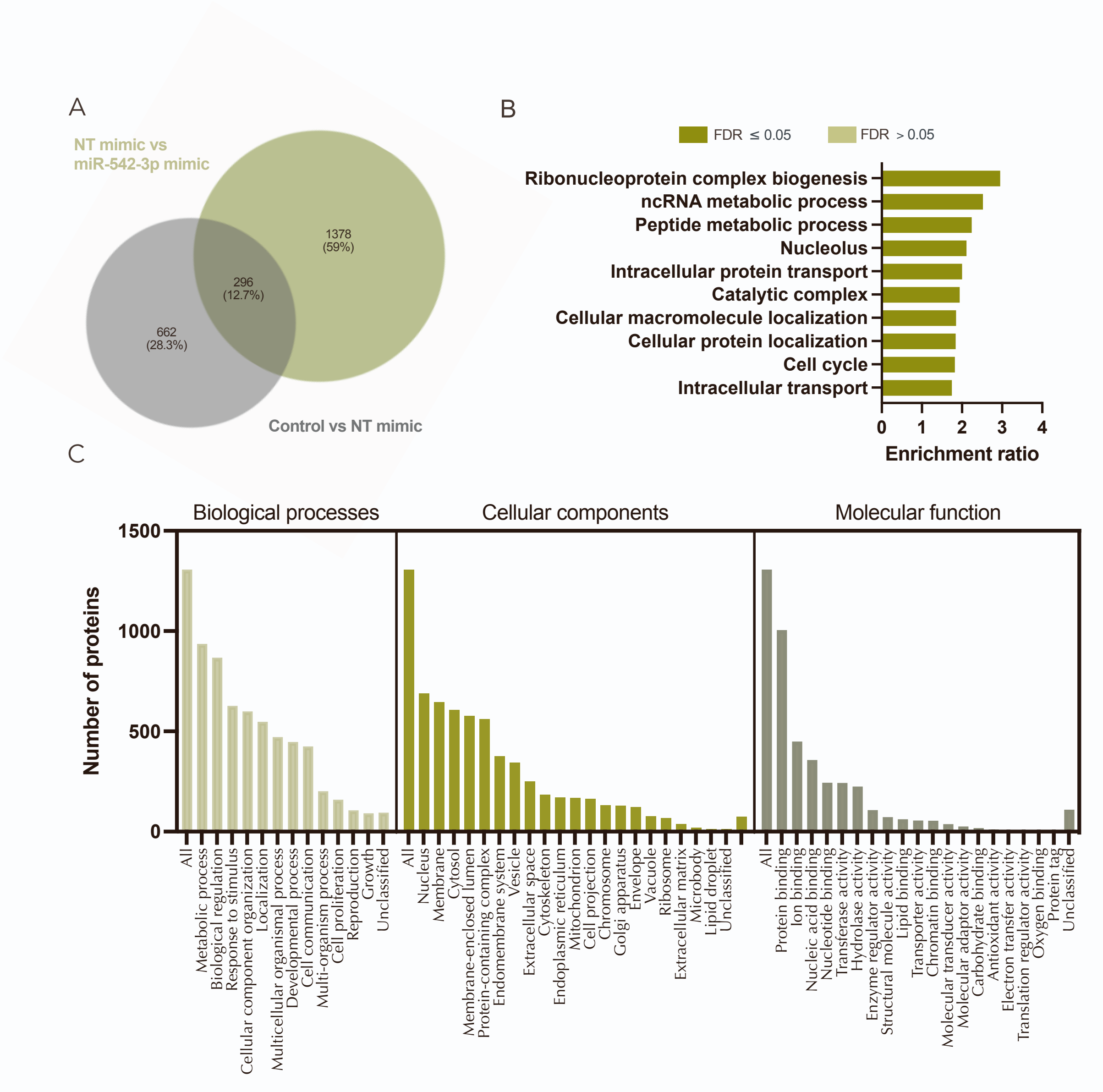

Supplementary Figure 4: *Proteins altered in Ishikawa cells following treatment with miR-542-3p mimic related to Figure 11.* **A)** Venn diagram depicting total number of significantly differentially expressed proteins (p<0.05) following transfection of Ishikawa cells (n=3 biological replicates) with miR-542-3p mimic (RHS) and non-targeting (NT) mimic (LHS). **B)** Enriched KEGG pathways associated with miR-542-3p mimic regulated proteins. **C)** WebGestalt overrepresentation analysis of biological process, cellular component, and

molecular function categories for identified significantly differentially expressed proteins (p<0.05) in response to miR-542-3p mimic.

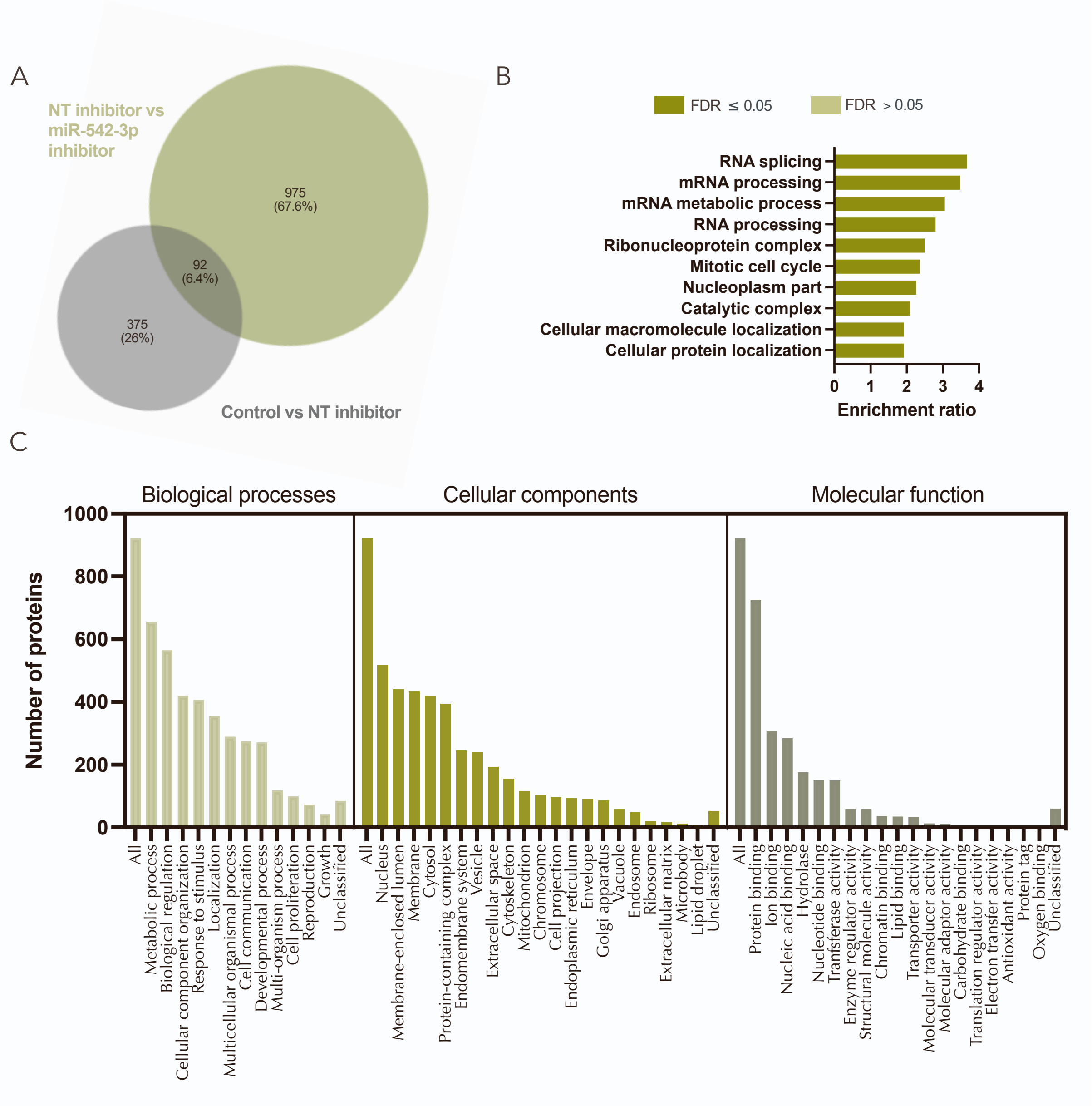

Supplementary Figure 5: *Proteins altered in Ishikawa cells following treatment with miR-542-3p inhibitor related to Figure 11.* **A)** Venn diagram depicting total number of significantly differentially expressed proteins (p<0.05) following transfection of Ishikawa cells (n=3 biological replicates) with miR-542-3p inhibitor (RHS) and non-targeting (NT) inhibitor (LHS). **B)** Enriched KEGG pathways associated with miR-542-3p inhibition regulated proteins. **C)**

WebGestalt overrepresentation analysis of biological process, cellular component, and molecular function categories for identified significantly differentially expressed proteins (p<0.05) in response to miR-542-3p inhibitor.

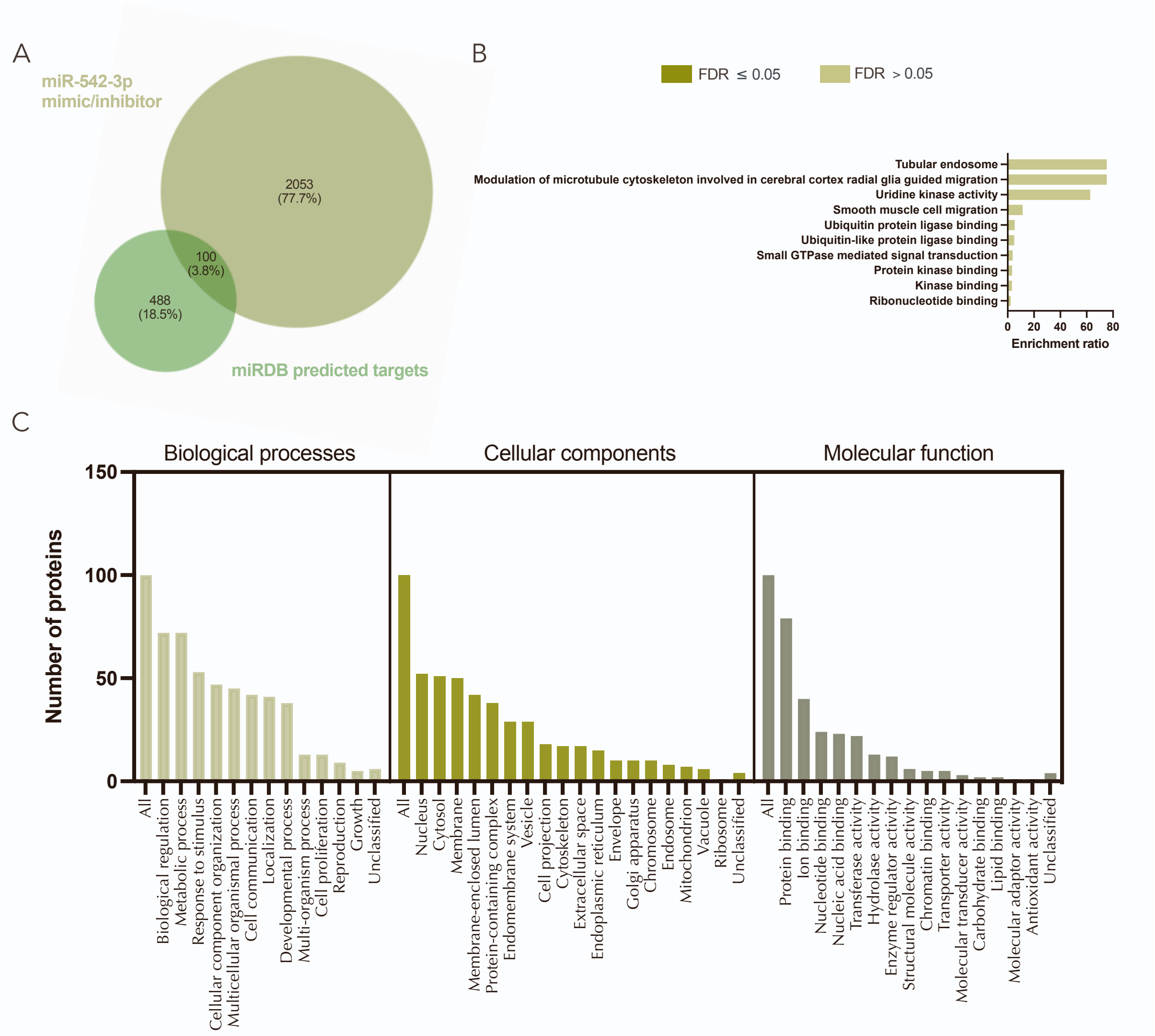

Supplementary Figure 6: *Proteins altered in Ishikawa cells following treatment with miR-542-3p mimic and inhibitor compared to mirDB predicted targets related to Figure 11.* **A)** Venn diagram depicting total number of significantly differentially expressed proteins (p<0.05) following transfection of Ishikawa cells (n=3 biological replicates) with miR-542-3p mimic or inhibitor vs non-targeting controls and mirDB predicted targets. **B)** Enriched KEGG pathways associated with miR-542-3p mimic and inhibition regulated proteins and predicted target

overlap. **C)** WebGestalt overrepresentation analysis of biological process, cellular component and molecular function categories for identified significantly differentially expressed proteins (p<0.05) in response to miR-542-3p mimic and inhibition and predicted targets.

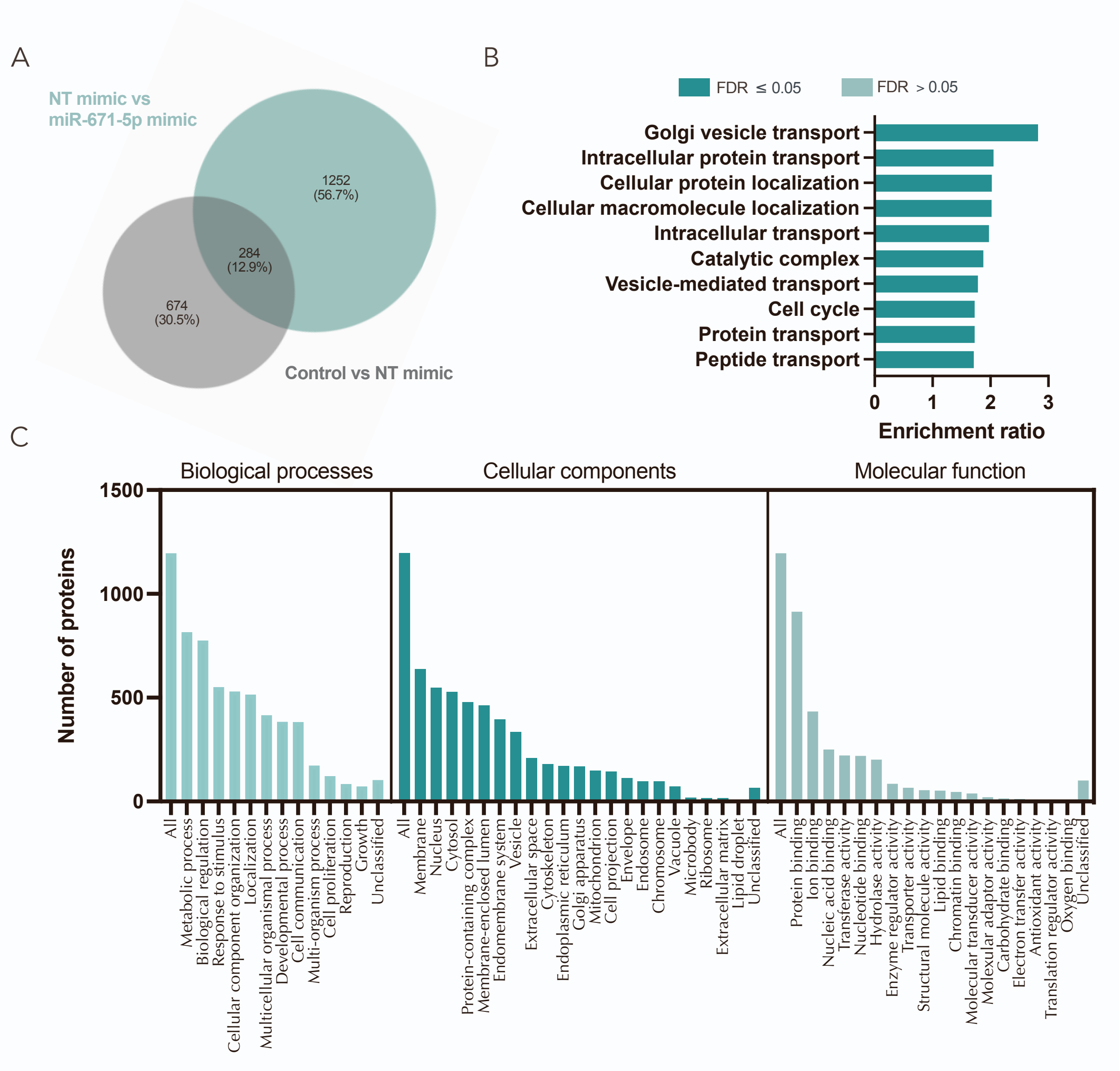

Supplementary Figure 7: *Proteins altered in Ishikawa cells following treatment with miR-671-5p mimic related to Figure 13.* **A)** Venn diagram depicting total number of significantly differentially expressed proteins (p<0.05) following transfection of Ishikawa cells (n=3 biological replicates) with miR-671-5p mimic (RHS) and non-targeting (NT) mimic (LHS). **B)** Enriched KEGG pathways associated with miR-671-5p mimic regulated proteins. **C)**

WebGestalt overrepresentation analysis of biological process, cellular component and molecular function categories for identified significantly differentially expressed proteins (p<0.05) in response to miR-671-5p mimic.

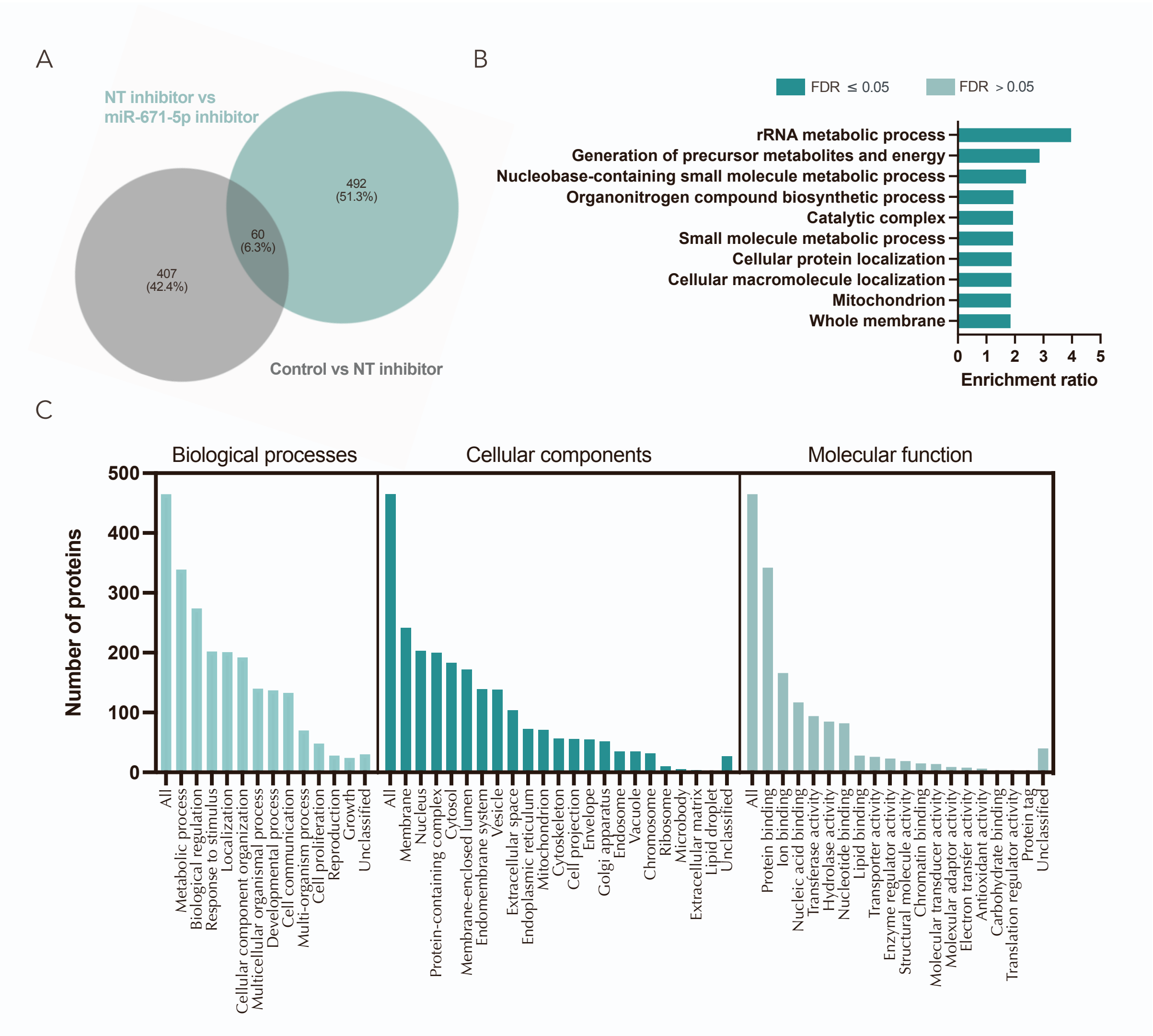

Supplementary Figure 8: *Proteins altered in Ishikawa cells following treatment with miR-671-5p inhibitor related to Figure 13.* **A)** Venn diagram depicting total number of significantly differentially expressed proteins (p<0.05) following transfection of Ishikawa cells (n=3 biological replicates) with miR-671-5p inhibitor (RHS) and non-targeting (NT) mimic (LHS). **B)** Enriched KEGG pathways associated with miR-671-5p inhibitor regulated proteins. **C)** WebGestalt overrepresentation analysis of biological process, cellular component and

molecular function categories for identified significantly differentially expressed proteins (p<0.05) in response to miR-671-5p inhibitor.

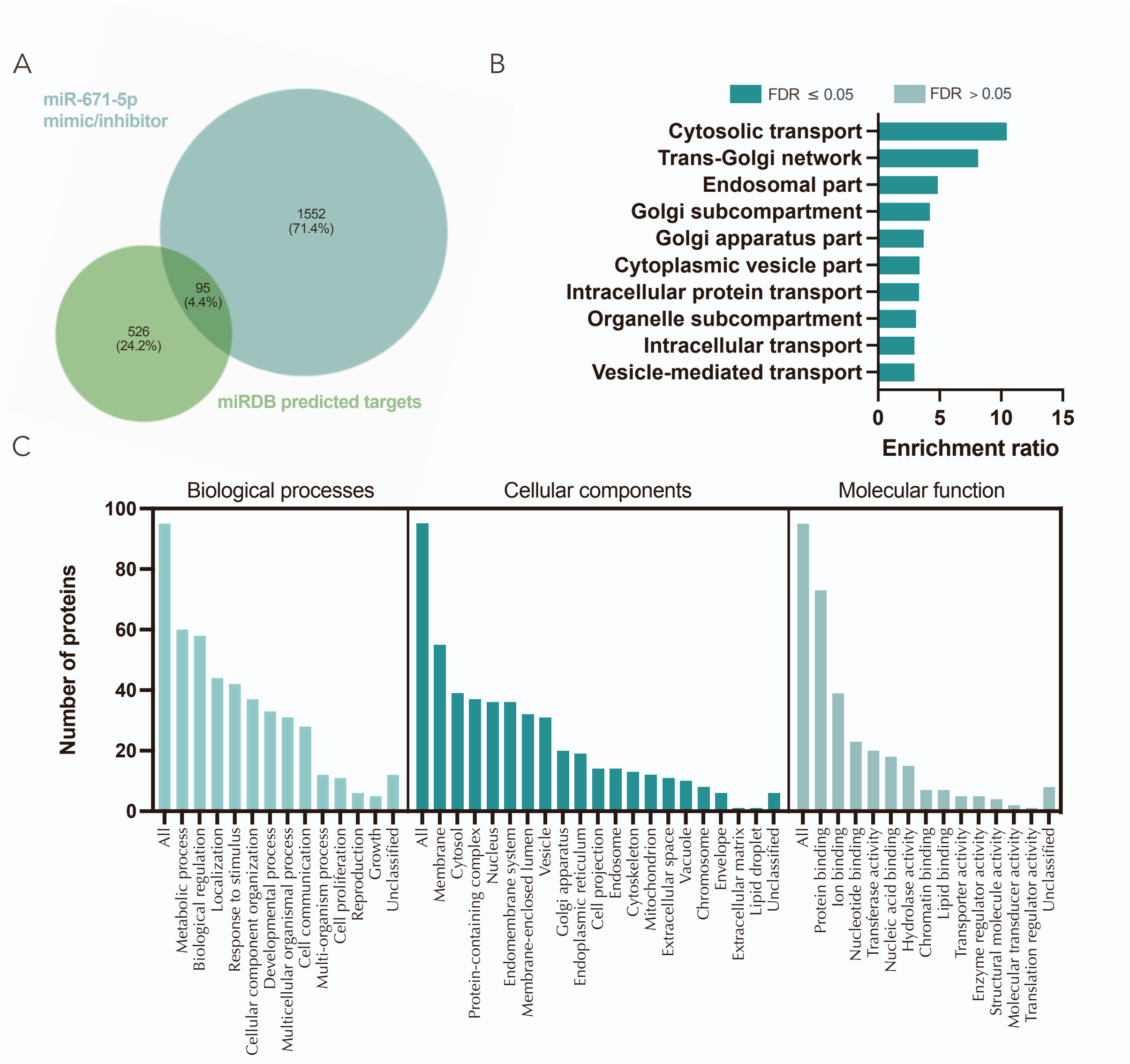

Supplementary Figure 9: *Proteins altered in Ishikawa cells following treatment with miR-671-5p mimic and inhibitor compared to mirDB predicted targets related to Figure 13.* **A)** Venn diagram depicting total number of significantly differentially expressed proteins (p<0.05) following transfection of Ishikawa cells (n=3 biological replicates) with miR-671-5p mimic or inhibitor vs non-targeting controls and mirDB predicted targets. **B)** Enriched KEGG pathways associated with miR-671-5p mimic and inhibition regulated proteins and predicted target

overlap. **C)** WebGestalt overrepresentation analysis of biological process, cellular component and molecular function categories for identified significantly differentially expressed proteins ($p<0.05$) in response to miR-671-5p mimic and inhibition and predicted targets.
